# A Multifunctional Therapeutic Strategy Using P7C3 as A Countermeasure Against Bone Loss and Fragility in An Ovariectomized Rat Model of Postmenopausal Osteoporosis

**DOI:** 10.1002/advs.202308698

**Published:** 2024-03-13

**Authors:** Fei Wei, Megan Hughes, Mahmoud Omer, Christopher Ngo, Abinaya Sindu Pugazhendhi, Elayaraja Kolanthai, Matthew Aceto, Yasmine Ghattas, Mehdi Razavi, Thomas J Kean, Sudipta Seal, Melanie Coathup

**Affiliations:** ^1^ Biionix Cluster University of Central Florida Orlando FL 82816 USA; ^2^ School of Biosciences Cardiff University Wales CF10 3AT UK; ^3^ College of Medicine University of Central Florida Orlando FL 32827 USA; ^4^ Advanced Materials Processing and Analysis Centre, Nanoscience Technology Center (NSTC) University of Central Florida Orlando FL 32826 USA

**Keywords:** bone loss, bone protection, fragility fracture, osteoporosis, ovariectomy, P7C3

## Abstract

By 2060, an estimated one in four Americans will be elderly. Consequently, the prevalence of osteoporosis and fragility fractures will also increase. Presently, no available intervention definitively prevents or manages osteoporosis. This study explores whether Pool 7 Compound 3 (P7C3) reduces progressive bone loss and fragility following the onset of ovariectomy (OVX)‐induced osteoporosis. Results confirm OVX‐induced weakened, osteoporotic bone together with a significant gain in adipogenic body weight. Treatment with P7C3 significantly reduced osteoclastic activity, bone marrow adiposity, whole‐body weight gain, and preserved bone area, architecture, and mechanical strength. Analyses reveal significantly upregulated platelet derived growth factor‐BB and leukemia inhibitory factor, with downregulation of interleukin‐1 R6, and receptor activator of nuclear factor kappa‐B (RANK). Together, proteomic data suggest the targeting of several key regulators of inflammation, bone, and adipose turnover, via transforming growth factor‐beta/SMAD, and Wingless‐related integration site/be‐catenin signaling pathways. To the best of the knowledge, this is first evidence of an intervention that drives against bone loss via RANK. Metatranscriptomic analyses of the gut microbiota show P7C3 increased *Porphyromonadaceae* bacterium, Candidatus Melainabacteria, and *Ruminococcaceae* bacterium abundance, potentially contributing to the favorable inflammatory, and adipo‐osteogenic metabolic regulation observed. The results reveal an undiscovered, and multifunctional therapeutic strategy to prevent the pathological progression of OVX‐induced bone loss.

## Graphical abstract

No available intervention definitively prevents or manages osteoporosis. In an ovariectomized rat model of postmenopausal osteoporosis, Pool 7 Compound 3 targets pivotal biomolecules involved in inflammation resolution, bone, and adipose homeostasis. Further, and within the gut microbiome, several anti‐inflammatory commensal species able to directly alter adipo‐osteogenic interactions are regulated. Results reveal a multifunctional therapeutic strategy to prevent the pathological progression of osteoporosis.

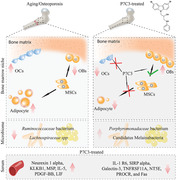

## 1 Introduction

Osteoporosis is a common, systemic, chronic, and progressive metabolic bone disease characterized by the gradual deterioration of bony architecture and composition, leading to decreased bone density and increased risk of fractures.^[^ ^1^ ^]^ Globally, osteoporosis affects a vast number of individuals and is considered a major public health concern, with women and the elderly being at a higher risk.^[^ ^1^, ^2^ ^]^ Approximately 200 million people worldwide live with osteoporosis and ≈8.9 million fragility fractures occur each year.^[^ ^3^ ^]^ In the United States alone, and in individuals >50 years of age, a reported 10.2 million Americans (≈2 million men^[^ ^4^ ^]^) are affected by osteoporosis,^[^ ^5^ ^]^ and a further 43.3 million have low bone mass.^[^ ^1^, ^6^ ^]^ Further, and due to our aging population, the number of osteoporotic related fragility fractures in the U.S. is projected to rise by 68% from the present mean of ≈1.9 million, to 3.2 million by 2040.^[^ ^2^ ^]^ Subsequently, the present associated costs of both acute and long‐term osteoporosis‐related bone treatment falls within $10 – 17 billion annually in the U.S,^[^ ^7^, ^8^ ^]^ is projected to rise to $25.3 billion in 2025,^[^ ^9^ ^]^ and will undoubtedly bear a huge health‐economic burden in all regions of the world.^[^ ^10^, ^11^ ^]^ Presently, no available pharmacological or non‐pharmacological interventions definitively manage or prevent osteoporosis, as all have their disadvantages. For example, the use of hormone replacement therapy with estrogen dates back to the 1940s^[^ ^12^ ^]^ and is still one of most commonly used therapies for osteoporosis prevention.^[^ ^13^, ^14^, ^15^ ^]^ However, it is not recommended for long‐term treatment due to its disputed potential to increase life‐threatening risks including breast or endometrial cancer, and cardiovascular disease.^[^ ^12^, ^16^, ^17^ ^]^ Bisphosphonates, such as alendronate, risedronate, and zoledronate, are also commonly used medications.^[^ ^18^, ^19^, ^20^, ^21^ ^]^ However, prolonged use is not advised due to an increased incidence of mandibular osteonecrosis, and atypical femoral fractures.^[^ ^21^ ^]^ Odanacatib, a cathepsin K inhibitor, inhibits osteoporotic bone loss,^[^ ^22^ ^]^ but is associated with significantly higher rates of atrial fibrillation and stroke.^[^ ^23^ ^]^ Denosumab is a humanized monoclonal antibody to receptor activator nuclear factor kappa‐B (NF‐κB) ligand (RANKL), thereby targeting the downregulation of osteoclast activity.^[^ ^13^ ^]^ Although as effective as bisphosphonates,^[^ ^24^ ^]^ similar adverse effects have been reported.^[^ ^25^ ^]^ More recently, Romozosumab, also a humanized monoclonal antibody, which binds and inhibits the action of sclerostin, was approved for clinical use in 2019.^[^ ^26^ ^]^ However, concerns including an increased incidence of cardiovascular and cerebrovascular events compared with alendronate, have been reported.^[^ ^27^ ^]^ Osteoporosis is closely related to epigenetics and emerging evidence highlights microRNAs,^[^ ^28^, ^29^, ^30^ ^]^ long non‐coding RNAs,^[^ ^31^, ^32^, ^33^ ^]^ and circular RNAs^[^ ^34^, ^35^, ^36^ ^]^ as significant epigenetic regulators involved in bone metabolism. This approach is considered a promising novel therapeutic strategy however, deeper insights into their roles will facilitate the development of efficient treatment strategies into the future.^[^ ^37^ ^]^ Thus, there is an urgent need to develop effective prevention and treatment strategies to mitigate this major public health issue.

At the cellular level, osteoporosis is primarily caused by an imbalance in the bone resorption versus formation response. This involves functional changes in the activity of mesenchymal stem cells within the bone marrow (BMSCs),^[^ ^38^ ^]^ including, BMSC senescence,^[^ ^39^ ^]^ and biased BMSC differentiation toward adipogenesis at the expense of osteoblastogenesis.^[^ ^40^ ^]^ Additionally, excessive osteoclastogenesis, and osteoclastic activity is observed,^[^ ^41^ ^]^ with osteocyte apoptosis and subsequent trabecular bone loss, microstructural deterioration and weakened bone also playing a crucial cellular role in inducing overall osteoporotic bone resorption.^[^ ^42^, ^43^, ^44^ ^]^ Moreover, recent studies have suggested that various immune cells, including macrophages, dendritic cells, neutrophils, eosinophils, natural killer cells, and the prolonged release of pro‐inflammatory mediators also contribute to the manifestation of osteoporosis.^[^ ^45^ ^]^ At the molecular level, the interaction between NF‐κB (also known as RANK or TNFRSF11A) and RANKL, play a crucial role in controlling the differentiation and survival of osteoclasts.^[^ ^46^ ^]^ RANKL, expressed on various cell surfaces including, osteoblasts, osteocytes, macrophages, and activated T cells, binds to the RANK receptor on osteoclasts and its precursors, leading to osteoclast maturation and bone resorption.^[^ ^47^ ^]^ During healthy turnover, osteoprotegerin (OPG) is a naturally occurring high affinity countermeasure that competitively binds to RANKL, and is thereby critical in regulating osteoclast maturation and subsequent bone resorption.^[^ ^4^ ^]^ Notably, an age‐dependent increase in the expression of RANKL, parallel to decreasing levels of OPG has been reported.^[^ ^48^ ^]^

At the molecular level, the neuroprotective and nicotinamide adenine dinucleotide (NAD^+^)/NAD + H (NADH)‐stabilizing aminopropyl carbazole Pool 7 compound 3 (P7C3), exerts its activity through activation of the intracellular rate‐limiting enzyme, nicotinamide phosphoribosyltransferase (NAMPT) involved in the NAD^+^ salvage pathway.^[^ ^49^, ^50^ ^]^ This pathway is required for ≈500 different enzymatic reactions crucial for maintaining cellular energy metabolism and redox balance.^[^ ^51^, ^52^ ^]^ Rodent and human studies have shown that the concentration of NAD^+^ declines with age,^[^ ^53^, ^54^ ^]^ and is associated with the age‐related decline in osteoprogenitor number and subsequently, bone mass.^[^ ^55^ ^]^ Further, increasing the levels of NAD^+^ has been shown to prevent age‐associated dysfunction including deoxyribonucleic acid (DNA) damage, mitochondrial dysfunction, and cell senescence.^[^ ^52^ ^]^ Recently, Lu and colleagues reported that nicotinamide mononucleotide supplementation, the precursor to NAD^+^, attenuated cellular senescence, partially prevented the progression of osteoporosis in an ovariectomized (OVX) model, and accelerated bone healing in osteoporotic mice.^[^ ^56^ ^]^ However, few other studies have directly investigated the role of NAD^+^ or its precursors as a countermeasure against osteoporosis. Notably, and when reduced, P7C3 treatment has been shown to normalize intracellular NAD^+^/NADH levels through without elevating NAD^+^ above normal physiological concentrations.^[^ ^50^, ^57^, ^58^, ^59^ ^]^ As such, this warrants further investigation.

Here, we investigated whether P7C3 can protect against bone loss induced by ovariectomy. Using an OVX‐induced bone loss model, our results reveal P7C3 inhibited osteoclastic activity, bone marrow adiposity, and whole‐body weight gain, leading to maintained bone area, architecture, and mechanical strength, despite ovariectomy. The mechanistic novelty is based on P7C3 significantly increasing serum protein levels of macrophage stimulating protein (MSP), interleukin‐5 (IL‐5), platelet derived growth factor‐BB (PDGF‐BB), leukemia inhibitory factor (LIF), with downregulation of interleukin‐1 (IL‐1) R6, Galectin‐3, and tumor necrosis factor receptor superfamily member 11a (TNFRSF11A orRANK), as well as tyrosine kinase receptor modulation (including, fibroblast growth factor receptor 3 (FGFR3), insulin‐like growth factor receptor‐1 (IGF‐1R), receptor‐to‐receptor tyrosine kinase (Ryk), and ephrin type‐B receptor 2 (EphB2)). Together, several pivotal biomolecules involved in inflammation resolution, bone, and adipose homeostasis, and osteoporosis were targeted. To the best of our knowledge, this is first evidence of an intervention that drives against bone loss by contributing to a reduction in the expression of RANK. Recently, the gut microbiome (GM) is emerging as a promising therapeutic target in the management of inflammatory, metabolic disorders, in regulating bone homeostasis,^[^ ^60^ ^]^ and primary osteoporosis.^[^ ^61^ ^]^ Notably, P7C3 increased the relative abundance of *Porphyromonadaceae* bacterium, an anti‐inflammatory bacterial species reportedly able to directly alter adipo‐osteogenic interactions within the host,^[^ ^62^ ^]^ and Candidatus Melainabacteria, also a lipid regulator.^[^ ^63^ ^]^ Levels of *Ruminococcaceae* bacterium, an important contributor to metabolic and immune function, and where low levels are associated with the elderly, frail,^[^ ^64^, ^65^ ^]^ as well as heightened osteoclastic activity in an OVX animal model, was also increased by P7C3.^[^ ^66^ ^]^

## 2 Results

### 2.1 Elemental Composition, and Chemical Analysis of P7C3 using X‐Ray Photoelectron Spectroscopy

**Figure 1:**
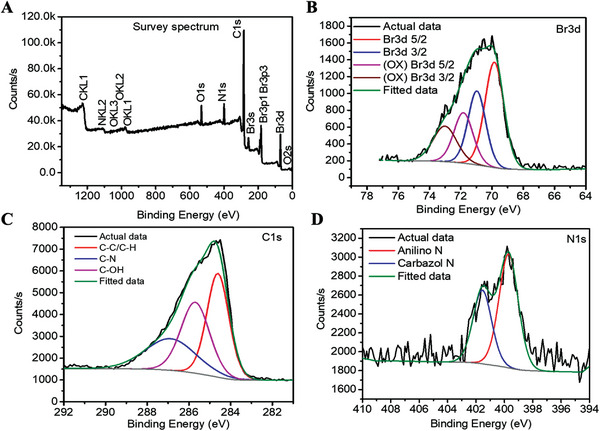
X‐ray photoelectron spectroscopy (XPS) analysis of P7C3 at room temperature. A] Bromide, carbon, nitrogen, and oxygen peaks were obtained in the XPS survey spectrum. B] Br 3d produced two bromide peaks (Br 3d 5/2, and Br 3d 3/2) and two oxidized bromide peaks ((OX) Br 3d 5/2, and (OX) Br 3d 3/2). C] The C1s signal produced three carbon peaks of C‐C/C‐H, C‐N and C‐OH. [D] The N1s signal identified the anilino N and carbazole N peaks.

P7C3 was subjected to X‐ray photoelectron spectroscopy (XPS) analysis at room temperature, within the binding energy region characteristic of the bromine (Br) 3d, carbon (C)1s, and nitrogen (N)1s orbitals (**Figure** **1**A–D). The Br 3D signals de‐convoluted into four different peak contributions; namely, the spin‐orbit coupling produced two Br peaks: Br 3d5/2 (69.85 eV), and Br 3d3/2 (70.97 eV). Additionally, two oxidized Br peaks were observed: (OX) Br 3d5/2 (71.83 eV), and (OX) Br 3d3/2 (73.01 eV). The carbon peak was de‐convoluted into three different peaks, which were assigned to C‐C/C‐H (284.65 eV), C‐OH (285.57 eV) and C‐N (286.57 eV) peaks. Similarly, the N1s peak was de‐convoluted into two peaks attributed to anilino N (399.67 eV) and carbazol N (401.56 eV) bonding. Further our calculations determined the C/Br, C/N and Br/N atomic percentage ratio was 9.95, 9.45 and 0.93 respectively, and similar to the theoretical value of P7C3 (C_21_H_18_Br_2_N_2_O), thus confirming the pure phase of P7C3 investigated.

### 2.2 P7C3 is Non‐Cytotoxic and Promotes hBMSC Osteogenesis via the TGF‐β/SMAD Signaling Pathway While Inhibiting Adipogenesis In Vitro

**Figure 2:**
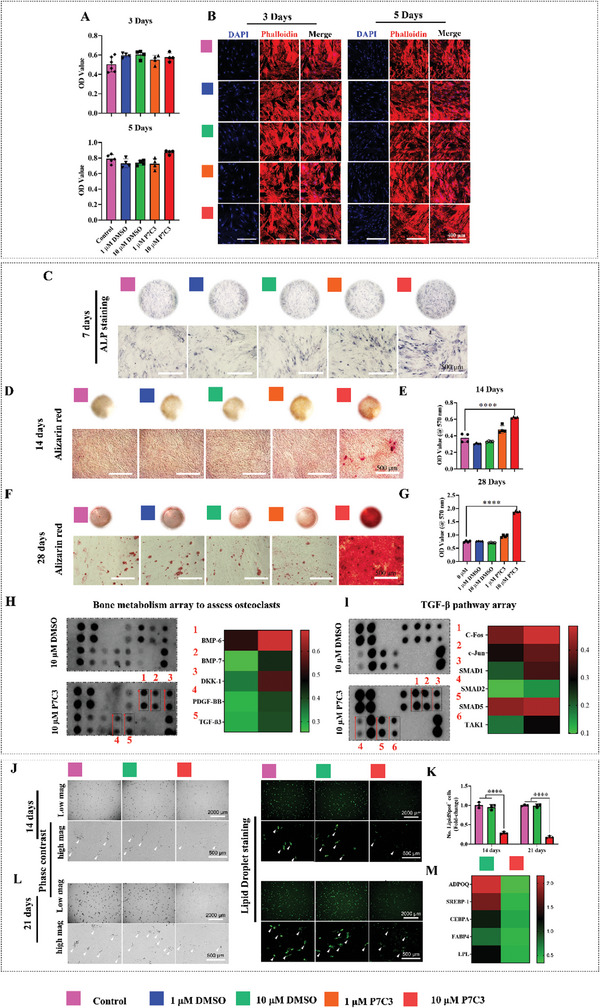
P7C3 is non‐cytotoxic and promotes hBMSC osteogenesis while inhibiting adipogenesis. A] The MTT assay was performed to determine cell metabolic activity. hBMSCs were treated with 0, 1 or 10 µM of P7C3 for 3 days and 5 days. 1 or 10 µM of DMSO were used as a vehicle control. B] Representative confocal microscopy images of hBMSCs treated with or without 10 µM of P7C3. The cells were fixed, stained with phalloidin (red) and DAPI (blue), and examined using confocal laser scanning microscopy. C] ALP staining results of hBMSCs cultured in osteogenic induction medium supplemented with P7C3 or DMSO vehicle (day 7). Representative ALP‐stained micrographs are shown in the lower panels of each figure. D] Alizarin red S staining for mineral deposition. Mineral deposition appears bright red in color (day 14). E] Quantification of mineralized calcium nodules (day 14). F] Alizarin red S staining results (day 28). Intense mineralized calcium nodule staining was noted following 10 µM P7C3 treatment. G] Quantification of mineralized calcium nodules (day 28). H] Results from a human bone metabolism array and quantification via a heat map. hBMSCs were either treated with 10 µM DMSO or 10 µM P7C3 in osteogenic differentiation media for 21 days. The cells were collected for array analysis. I] A human TGF‐β pathway phosphorylation array and quantification via a heat map. hBMSCs were either treated with 10 µM DMSO or 10 µM P7C3 in osteogenic differentiation media for 1 h. The cells were collected for array analysis. J–L] Representative phase contrast and LipidSpot™ Lipid Droplet‐stained images of hBMSCs cultured in adipogenic induction medium supplemented with P7C3 or the DMSO vehicle for 14 days J] and 21 days L]. The quantification of LipidSpot™^+^ cells is presented in image K. M] Heat map of mRNA expression levels of adipogenesis‐related genes were measured after 12 days of culture. hBMSCs were cultured in adipogenic induction medium supplemented with either P7C3 or DMSO for 12 days. The gene expression of *ADPOQ*, *SREBP‐1*, *CEBPA*, *FABP4*, and *LPL* were investigated. *p < 0.05, **p < 0.01, ****p < 0.0001.

Using a MTT assay, we first investigated the effect of P7C3 on the metabolic activity of human BMSCs (hBMSCs), thereby assessing cell viability, proliferation, and cytotoxicity. As shown in **Figure** **2**A, our results show that both concentrations of P7C3 (1 and 10 µM) were non‐toxic and cells displayed comparable levels of activity after 3 and 5 days of culture when compared to the untreated control group. The safety of this dose is supported by unpublished data where a range of doses were investigated, and a similar result was also obtained in our previous study.^[^ ^67^ ^]^ P7C3‐induced alterations within the cytoskeletal morphology of hBMSCs was assessed via phalloidin and DAPI staining (Figure 2B). On days 3 and 5, hBMSCs treated with 10 µM of P7C3 showed a similar morphology and actin filament content compared to the control group, indicating that 10 µM of P7C3 did not modify hBMSC cytoskeletal morphology.

To examine the effect of P7C3 on hBMSC osteogenic differentiation in vitro, both the levels of alkaline phosphatase (ALP) expression and bone mineral deposition were evaluated. Qualitative analysis revealed more pronounced ALP staining in the group treated with 10 µM of P7C3 compared to the non‐treated control and dimethyl sulfoxide (DMSO) vehicle‐control groups on day 7 (Figure 2C). To support this, our results revealed that hBMSCs treated with 10 µM P7C3 exhibited increased mineralization, as evidenced by the intensified staining of calcium‐rich deposits (red staining) and a significant increase (*p* < 0.0001) in the amount of mineralized nodules compared to the control group following 14 days of culture (Figure 2D and E). Moreover, quantitative analyses further revealed a >3‐fold increase in bone mineral deposition following the administration of 10 µM of P7C3 to hBMSCs, and at 28 days compared to the control group (*p* < 0.0001). These findings show that P7C3 has the potential to significantly upregulate both hBMSC osteogenic differentiation, as well as the deposition of bone mineral by mature osteoblasts (Figure 2F,G).

To further investigate the action of P7C3 on osteoblastic function and activity, a human bone metabolism array was used to evaluate the expression of osteogenesis‐related markers. Our results reveal that hBMSCs treated with 10 µM P7C3 exhibited upregulation of proteins; bone morphogenetic protein 6 (BMP‐6), BMP‐7, Dickkopf‐1 (DKK‐1), PDGF‐BB, and transforming growth factor‐beta 3 (TGF‐β3) (Figure 2H). Further, a protein microarray analysis of the cell lysate was used to determine TGF‐β phosphorylation pathway activity. Six phosphorylated proteins were evaluated, and data indicated an upregulation of c‐Fos, c‐Jun, SMAD1, SMAD2, SMAD5, and TGF‐β‐activated kinase 1 (TAK1) following 10 µM of P7C3 treatment (Figure 2I).

Controlling the adipo‐osteogenic lineage commitment of BMSCs in favor of osteogenesis at the expense of adipogenesis, is considered a promising new strategic approach to promote bone regeneration and repair. However, the underlying mechanisms involved are yet to be clarified. To study the impact of P7C3 on the adipogenic differentiation of hBMSCs in vitro, lipid droplet staining was used to assess adipocyte formation. Following 14 days of culture, the results shown in Figure 2J and K demonstrate a similar quantity of adipocyte formation in the control and DMSO vehicle‐control groups, while a significant reduction in adipocyte formation was observed in the 10 µM P7C3 treated group compared to control samples (*p* < 0.001). In addition, and on day 21, hBMSCs that received no P7C3 treatment displayed pronounced lipid droplet formation, whereas the group treated with 10 µM P7C3 showed a considerable, and significant decrease in staining (*p* < 0.001) (Figure 2L,K).

To further validate the results obtained from lipid droplet staining, qRT‐PCR was performed to detect the expression levels of adipogenesis‐related genes, including, *adiponectin* (*ADPOQ)*, and *sterol regulatory element‐binding protein 1 (SREBP‐1), CCAAT/enhancer‐binding protein alpha (CEBPA), fatty acid binding protein 4 (FABP4), lipoprotein lipase (LPL)*, and *peroxisome proliferator‐activated receptor‐gamma (PPAR* **γ**) (Figure 2M). Our results showed a significant decrease in the expression levels of *ADPOQ* (*p* < 0.0001), *SREBP‐1* (*p* < 0.01), *CEBPA* (*p* < 0.01), *FABP4* (*p* < 0.05), and *LPL* (*p* < 0.0001) in hBMSCs treated with P7C3 compared to the DMSO‐vehicle control group. Notably, at the time point and dose investigated, P7C3 did not significantly reduce *PPAR* **γ** expression (Figure S1, Supporting Information). Collectively, these findings suggest the DMSO‐vehicle control group upregulated adipogenesis while, notably, and under normal culture conditions, treatment with P7C3 led to increased hBMSC osteogenic differentiation parallel to a decrease in adipogenic differentiation. This was evidenced by an increased ALP staining and mineralization capability, as well as decreased lipid droplet staining and expression of adipogenesis‐related genes.

### 2.3 P7C3 Activates Multiple Receptor Tyrosine Kinases

**Figure 3:**
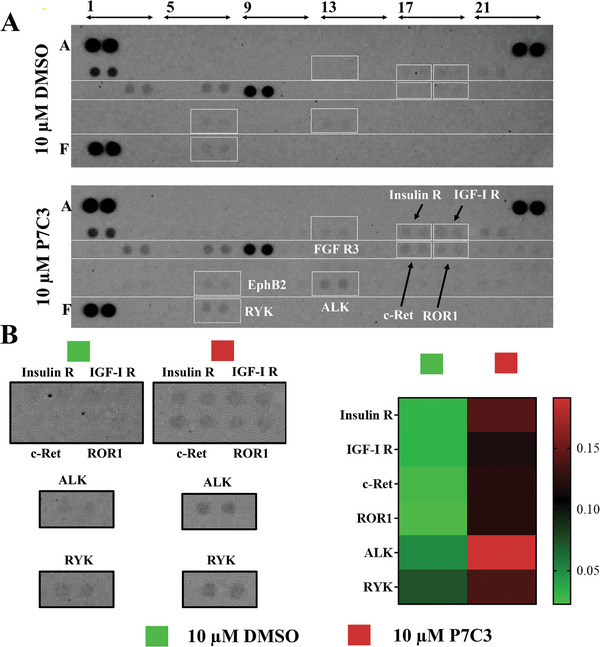
Phospho‐RTK array results demonstrate that P7C3 activates the phosphorylation state of multiple RTKs in hBMSCs. A] Phospho‐RTK array analysis of hBMSC cell lysates. hBMSCs were either treated with 10 µM DMSO or 10 µM P7C3 for 10 min. Cells were harvested for Phospho‐RTK array analysis. B,C] Quantification of Phospho‐RTK array is visualized via a heat map.

Protein tyrosine phosphorylation, which occurs mainly through the action of receptor tyrosine kinases (RTKs), is a fundamental cell‐to‐cell mediated regulatory mechanism controlling healthy cell proliferation, differentiation, communication, and adhesion.^[^ ^68^ ^]^ Protein tyrosine phosphorylation tightly modulates the lineage commitment of BMSCs,^[^ ^69^ ^]^ as well as influences the proliferation, osteogenic differentiation,^[^ ^70^ ^]^ and maturation of osteoblast precursors, as well as the function of osteocytes, and osteoclasts.^[^ ^71^, ^72^, ^73^ ^]^ Their deletion results in severe osteopenia, limb deformity, and impaired mineralization.^[^ ^74^, ^75^ ^]^ To further investigate the mode of action, we investigated the response of these cell surface proteins to P7C3 in vitro (**Figure** **3**). Our results show upregulation of insulin receptor (Insulin R), IGF‐1R, c‐Ret, Receptor Tyrosine Kinase Like Orphan Receptor 1 (ROR1), ALK receptor tyrosine kinase (ALK), EphB2, and Ryk.

### 2.4 P7C3 Inhibits Osteoclast Maturation, and Osteoclastic Activity In Vitro

**Figure 4:**
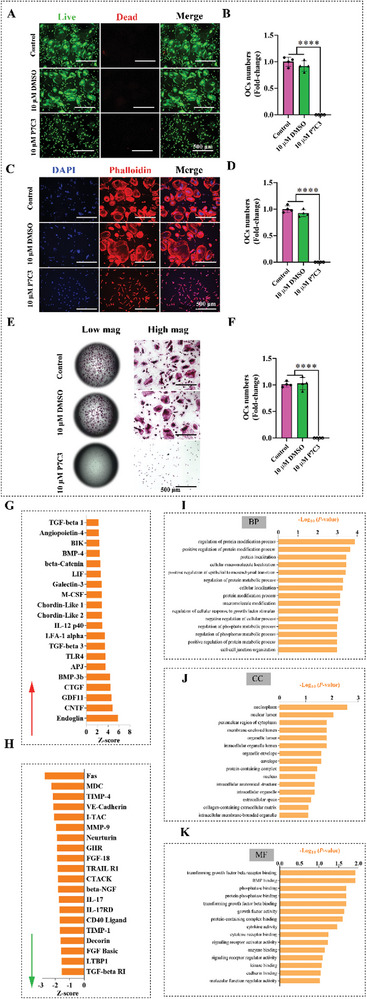
P7C3 attenuates osteoclastic maturation and activity in vitro. A] Representative confocal micrographs of human osteoclast precursors cultured with either DMSO or P7C3 over a 6‐day period. Prior to examination via confocal laser scanning microscopy, the cells were subjected to dual staining with fluorescein diacetate (live cells in green) and propidium iodide (dead cells in red). B] Quantitative analysis of mature osteoclast numbers using data derived from live/dead staining. ****p < 0.0001. C] Representative confocal microscopy images showing human osteoclast precursors cultured with either DMSO or P7C3 over a 6‐day period. Following fixation, the cells were stained with phalloidin (red) and DAPI (blue). D] Quantitative analysis of osteoclast numbers using data derived from phalloidin and DAPI staining. ****p < 0.0001. E] Representative images of TRAP staining showing multinucleated active osteoclasts (red). F] The number of osteoclasts were further quantitated via TRAP staining. ****p < 0.0001. G] The top 20 differentially expressed proteins (upregulated) following P7C3 treatment are presented. H] The top 20 differentially expressed proteins (downregulated) following P7C3 treatment are presented. I–K] GO enrichment analysis for differentially expressed biomarkers. BP = biological process I]; CC = cellular component J]; MF = molecular function K].

To explore the effect of P7C3 on osteoclast activities in vitro, we treated human osteoclast precursors with either 10 µM DMSO or 10 µM P7C3 for 6 days. We first performed a live/dead assay and our results showed that both 10 µM DMSO and 10 µM P7C3 exhibited a non‐toxic effect on human osteoclast precursors, where cells exclusively displayed a green color, indicating their viability (**Figure** **4**A). As shown in Figure 4B, the number of precursor cells able to develop into mature osteoclasts was significantly impeded following P7C3 treatment, and in contrast to both the control and 10 µM DMSO groups (*p* < 0.0001 in both cases). Results from phalloidin and DAPI staining showed the formation of multinucleated osteoclasts featuring a prominent F‐actin ring structure, in both the control and 10 µM DMSO groups. However, this distinctive structure was absent after treatment with 10 µM P7C3 (Figure 4C). Quantitative analysis revealed a significant reduction in mature osteoclast numbers following P7C3 treatment (*p* < 0.0001, Figure 4D). Representative phase contrast images showing the inhibitory effect of P7C3 on osteoclast maturation are shown in Figure S2 (Supporting Information).

Furthermore, within the 10 µM P7C3‐treated group, no tartrate‐resistant acid phosphatase‐positive (TRAP^+^) cells (activated osteoclasts) were detected (Figure 4E). In contrast, the control and 10 µM DMSO groups displayed elevated osteoclastic activity as evidenced by robust TRAP staining. Quantitative analysis showed a significant reduction in TRAP^+^ cell numbers following P7C3 treatment (*p* < 0.0001, Figure 4F).

At the end of the study, the supernatant was collected, and its cytokine and chemokine proteomic profile determined. Figure 4G and H show the 20 proteins that exhibited the most significant upregulation and the 20 proteins showing the most significant downregulation after treatment with P7C3. Data reveal increased abundance of Fas cell surface death receptor (Fas), macrophage‐derived chemokine (MDC), TIMP metallopeptidase inhibitor 4 (TIMP‐4), VE‐Cadherin, and interferon‐inducible T‐cell alpha chemoattractant or C‐X‐C motif chemokine 11 (I‐TAC). The top five proteins that were most reduced in expression were endoglin, ciliary neurotrophic factor (CNTF), growth and differentiation factor 11 (GDF11), connective tissue growth factor (CTGF), and bone morphogenetic bone 3b (BMP‐3b/GDF10). The proteins were also hierarchically clustered, and Figure 4I–K show the top 15 gene enrichment (GO) terms in the biological processes (BP), cellular compartment (CC), and molecular function (MF) categories, respectively. The top 5 upregulated GO terms of BPs were, regulation of protein modification process, positive regulation of protein modification process, protein localization, cellular macromolecule localization, and positive regulation of epithelial to mesenchymal transition. The top 5 CC terms were nucleoplasm, nuclear lumen, perinuclear region of cytoplasm, membrane‐enclosed lumen, and organelle lumen. Finally, the top 5 MF terms were transforming growth factor beta receptor binding, BMP binding, phosphatase binding, protein phosphatase binding, and transforming growth factor bet binding.

### 2.5 P7C3 is Non‐Toxic In Vivo and Reduces Osteoclast Activity and Bone Loss While Maintaining Biomechanical Strength in an OVX‐Induced Osteoporotic Animal Model

**Figure 5:**
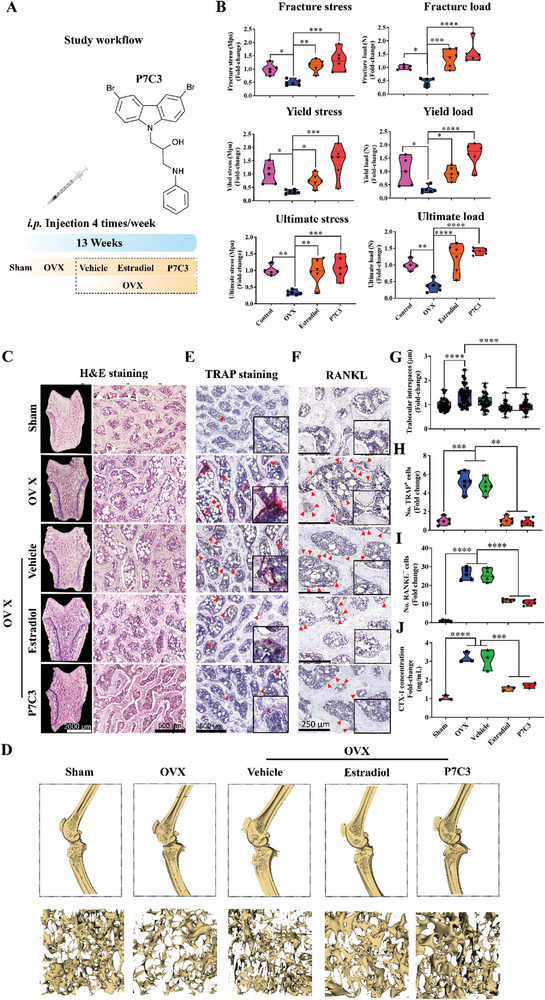
P7C3 (20 mg k^−1^g, given *i.p*.) administration maintains healthy levels of biomechanical bone strength and reduces bone loss, osteoclastogenesis, and activated osteoclastic activity in an OVX‐induced osteoporotic animal model. A] A flow chart of the animal experiment. B] The biomechanical properties of fracture load, fracture stress, yield load, yield stress, ultimate load, and ultimate stress at the tibial mid‐point during 3‐point bending analyses. Tibiae from OVX animals are biomechanically weaker, while animals receiving 20 mg k^−1^g *i.p*. treatment of P7C3 showed a significant increase in strength at 13 weeks post‐treatment. C] Representative H&E‐stained histology images of transverse sections through the femoral condyle. Images were acquired at ×2 (left panel) and ×10 (right panel) magnification, respectively. D] Tibiae and femora in each group were microCT scanned and 3D reconstructed images created using 3D Slicer. Representative images show trabecular bone loss in the OVX and DMSO‐vehicle groups, with an increased cancellous volume in animals treated with either Estradiol or P7C3. E] The TRAP activity of active osteoclasts was measured via TRAP staining. TRAP^+^ osteoclasts on the bone surface stained a purple‐red color and are indicated by red arrows. F] Immunohistochemical analysis of RANKL within trabecular bone in the distal femur. RANKL^+^ cells are shown by the red arrows. G] Quantification of trabecular interspaces. ****p < 0.0001. H] Quantification of TRAP^+^ osteoclasts per unit of bone surface (cells mm^−2^) via bone histomorphometric analyses (*n* = 6). I] Quantification of RANKL^+^ cells/bone surface (N mm^−1^). J] Serum levels of the bone resorption marker, CTX‐1 was analyzed using ELISA. P7C3 treatment significantly reduced the level of CTX‐1. *p < 0.05, **p < 0.01, ***p < 0.001, ****p < 0.0001.

Having established the favorable impact of P7C3 to cells in vitro, we next evaluated the effects of P7C3 in vivo using an OVX‐induced osteoporotic animal model (**Figure** **5**A). As shown in Figures S3–S7 (Supporting Information), our results reveal no histological indications of P7C3‐induced toxicity in the brain, kidney, liver, lung, or spleen following 13‐weeks of treatment.

Postmenopausal osteoporosis is characterized by a loss in biomechanical strength and an increase in bone fragility. To assess the effect of P7C3 treatment on ultimate stress, yield stress, and fracture stress following the onset of OVX‐induced osteoporosis, we performed a 3‐point bending test on rat tibiae. Figures S8–S10 (Supporting Information) shows the results for storage modulus (E’), loss modulus (E’’), and loss tangent (δ) in the different experimental groups, and at frequencies of 1, 5, and 10 Hz. Our data reveals that ovariectomy led to a significant reduction in fracture stress (*p* < 0.05), yield stress (*p* < 0.05), and ultimate stress (*p* < 0.01) when compared to the healthy, control group (Figure 5B). However, treatment with P7C3 maintained these parameters to similar levels as the control, and Estradiol‐given group, thereby displaying a significant increase in fracture stress (*p* < 0.001), yield stress (*p* < 0.0001), and ultimate stress (*p* < 0.0001) when compared to the OVX group. This shows that P7C3 administration prevents the OVX‐induced decline in the mechanical properties of the tibia during the 13‐week period post‐ovariectomy.

To investigate the potential effects of P7C3 on OVX‐induced pathological bone loss, skeletal changes were further assessed via Hematoxylin and Eosin (H&E)‐stained histological sectioning. As shown in Figure 5C,G, at 13 weeks post‐OVX, rats displayed significant bone loss, as characterized by increased bone marrow fat vacuole infiltration, thinner and shorter trabeculae, and reduced trabecular connectivity. In contrast, P7C3‐treated rats showed substantial resistance to OVX‐induced bone loss, with a bony structure similar to that of the sham control, and Estradiol‐given groups. These alterations in structure were supported following microCT analyses and 3D reconstruction (Figure 5D). To further understand the mechanism of action, we evaluated the effects of P7C3 on osteoclastic activity, and osteoclastogenesis via TRAP staining, RANKL immunohistochemical (IHC) staining, and serum type I collagen crosslinked C‐telopeptide (CTX‐1) protein levels. Transverse sections through the center of the femoral condyle in each group were prepared, and results revealed a significant increase in the presence of TRAP^+^ cells, and thus activated mature osteoclasts in the OVX and DMSO‐vehicle groups, while the number of active osteoclasts was significantly reduced in the P7C3‐treated, and Estradiol‐given animals (*p* < 0.0001 in both groups; Figure 5E,H). No significant difference in the number of TRAP^+^ osteoclasts was found when Estradiol‐ and P7C3‐treated rats were compared.

Osteoclastogenesis is promoted by RANKL, an essential signaling molecule that binds to its receptor (RANK) on osteoclast precursors, thereby initiating osteoclast differentiation and ultimately, bone resorption.^[^ ^76^ ^]^ To investigate the effects of P7C3 on RANKL expression as OVX‐induced osteoporosis progresses, we performed RANKL IHC staining on sections prepared through the femoral condyle. We found that the number of RANKL^+^ cells was significantly increased in the OVX and DMSO‐vehicle groups when compared with sham animals (*p* < 0.0001 in all groups, Figure 5F,I). However, and comparable to Estradiol‐given animals, P7C3 treatment resulted in a significant >3.5‐fold decrease in RANKL^+^ cells when compared to both the OVX and DMSO‐vehicle groups (*p* < 0.0001 in both cases). Finally, the protein CTX‐1 is released during collagen degradation and is used as a measure of osteoclastic activity, the overall level of systemic bone resorption,^[^ ^77^ ^]^ and as a clinical indicator of the response of therapy to postmenopausal osteoporosis.^[^ ^78^ ^]^ Our results show serum levels of CTX‐1 were also significantly reduced following P7C3 treatment and when compared to the OVX, and DMSO‐vehicle groups (*p* < 0.0001 in both cases; Figure 4J). Together, these findings show P7C3 may in part, exert its therapeutic effects against OVX‐induced bone loss by inhibiting bone resorption through the regulation of RANKL expression, and thus inhibiting osteoclastogenesis, and by reducing the number of mature and activated osteoclasts, which is consistent with our in vitro results.

### 2.6 P7C3 Attenuates OVX‐Induced Weight Gain, Bone Marrow Adiposity, and Visceral, and Subcutaneous Adipose Tissue

**Figure 6:**
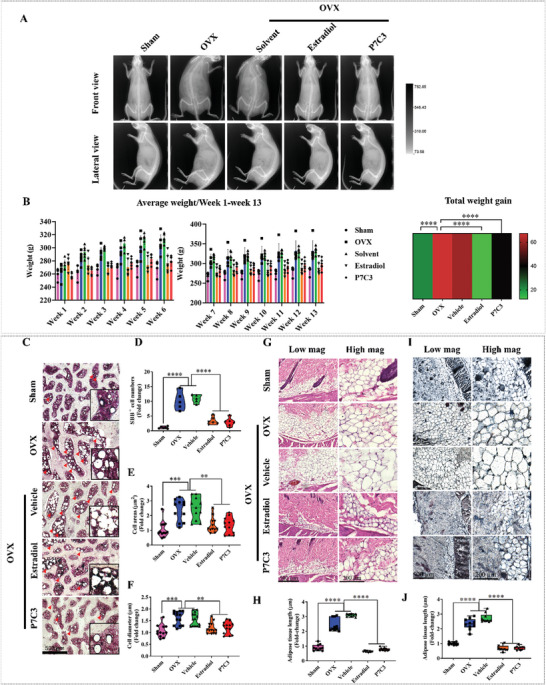
P7C3 treatment (20 mg k^−1^g) attenuates adipogenesis in an OVX‐induced osteoporotic animal model. A] Representative in vivo imaging photograph showing changes in body mass, with OVX and vehicle‐treated rats exhibiting significant increases in body mass compared to the sham control. However, rats treated with Estradiol or P7C3 showed reduced whole‐body mass, as observed using the Bruker In‐Vivo Xtreme system. B] Cumulative change in body weight, indicating a significant reduction in body weight between animal groups following Estradiol and P7C3 treatment. Statistically significant differences in OVX versus Estradiol groups: Week 3, 7, 8, **p < 0.01; Week 9, ***p < 0.001.; Week 4, 5, 6, 10, 11, 12, 13, ****p < 0.0001. Statistically significant differences in OVX versus P7C3 groups: Week 3, 4, 5, 7, 8, 11, *p < 0.05; Week 2, 9, 10, **p < 0.01.; Week 6, 12, 13, ***p < 0.001. A heat map of total weight gain (g) over the study is also presented. Statistically significant differences in Sham versus OVX groups, OVX versus Estradiol groups, and OVX versus P7C3 groups were measured. C] Representative images of Sudan Black B staining showing the presence of black‐colored adipocytes (red arrow). The nuclei were counterstained with nuclear fast red (red). D] Quantification of Sudan Black B^+^ cell number in each group. E] Quantification of Sudan Black B^+^ cellular area, and F] cell diameter. G,H] Representative H&E‐stained sections of subcutaneous adipose tissue at 13 weeks post‐injection were analyzed. Results show the presence of multiple layers of adipocytes in the OVX and DMSO‐vehicle‐treated groups, while adipose tissue thickness was significantly reduced following treatment with Estradiol and P7C3, showing no difference compared to the sham group (****p < 0.0001). I,J] Representative images of Sudan Black B staining of subcutaneous adipose tissue at 13 weeks post‐injection. Results show similar results as the H&E staining, indicating that the adipocytes were greatly reduced following treatment with Estradiol and P7C3. *p < 0.05, **p < 0.01, ***p < 0.001, ****p < 0.0001.

The whole‐body mass of animals in each group were imaged using an Xtreme II In Vivo imager (Bruker, Billerica, MA). Representative gross body mass images are displayed in Figure S11 (Supporting Information) and show the OVX and DMSO‐vehicle‐treated groups displayed the highest body mass, and cumulative weight gain when compared to the sham, Estradiol, and P7C3 groups over the 13‐week study period. As shown in **Figure** **6**A, rats in the OVX‐ and vehicle‐treated groups demonstrated an overall higher whole‐body mass at the end of the 13‐week period. In contrast, animals who received 4 days/week of Estradiol or P7C3 treatment, showed a marked decrease in overall cumulative weight gain, with no obvious qualitative differences observed when compared to the natural weight gain displayed in the healthy sham group. Body weight was also quantitatively assessed and recorded weekly (Figure 6B), and results show that on week 2 after treatment, mean body weight in the OVX and vehicle‐treated groups were 284.5 ± 9.2 g (*p* < 0.01), and 293.7 ± 9.1 g (*p* < 0.0001), respectively, which is significantly higher and a ≈8‐, and 12‐fold gain compared to the sham group (262.6 ± 9.5 g). While in contrast, the OVX animals treated with Estradiol (273.5 ±13.0 g) and P7C3 (265.3± 5.9 g) displayed a significantly reduced mean body weight at this early time point (both *p* < 0.01), and a similar weight to the sham group. This trend continued, and at the 13‐week post‐treatment time point, the OVX animals treated with Estradiol (289.0 ±16.9 g, *p* < 0.0001) and P7C3 (297.8 ±13.7 g, *p* < 0.001) both presented with a significantly lower and respective ≈11‐, and 8‐fold decrease in mean body weight when compared with the OVX animals (322.2 ±26.0 g). No significant differences were found when OVX control and vehicle‐treated (334.3 ± 9.3 g) animals were compared. Furthermore, OVX animals treated with Estradiol or P7C3 exhibited a significant (*p* < 0.001 in both cases) decrease in total weight gain over the course of the study (Figure 6B).

To assess the impact of P7C3 treatment on bone marrow adiposity, we utilized Sudan black B staining, which detects the presence of lipids including neutral triglycerides, phospholipids, and sterols. The results shown in Figure 6C demonstrate that the bone marrow of the sham group displayed minimal Sudan black B^+^ adipocytes, while there was a significant increase in bone marrow adiposity in the OVX group at 13 weeks. This was evidenced by increased adipocyte density (Figure 6D, ≈10‐fold increase, *p* < 0.0001), adipocyte area (Figure 6E, ≈2.3‐fold increase, *p* < 0.001), and adipocyte diameter (Figure 6F, ≈1.6‐fold increase, *p* < 0.001). However, treatment with P7C3 significantly reduced bone marrow adiposity, as shown by a substantial decrease in adipocyte number (*p* < 0.0001), adipocyte area (*p* < 0.01), and adipocyte diameter (*p* < 0.01) when compared to the OVX group. Further, the amount of visceral adipose tissue present was measured (weight (g)), and our results show that both Estradiol and P7C3 treated animals exhibited a significant reduction in visceral fat tissue compared to the OVX and DMSO‐vehicle groups (both *p* < 0.001) (Figure S12, Supporting Information). In addition, there was no significant difference in bone marrow adiposity between Estradiol‐treated and P7C3‐treated groups. The volume and thickness of subcutaneous adipose tissue was further investigated, with the OVX and vehicle‐treated group (both *p* < 0.0001) both demonstrating a significantly higher volume and thickness compared to the sham group. While in contrast, treatment with Estradiol and P7C3 resulted in a significant (both *p* < 0.0001) decrease in the volume and thickness of subcutaneous adipose tissue (Figure 6G,H). Consistent with the H&E staining result, Sudan black B staining yielded similar results (Figure 6I,J). Collectively, these findings show that P7C3 has a protective effect in reducing bone marrow adiposity, as well as subcutaneous and visceral adipose tissue levels in OVX‐induced osteoporotic rats.

### 2.7 P7C3 Induces Alterations in Serum Cytokine/Chemokine Protein Profiles When Compared to Control, and Estradiol‐Given Animals

**Figure 7:**
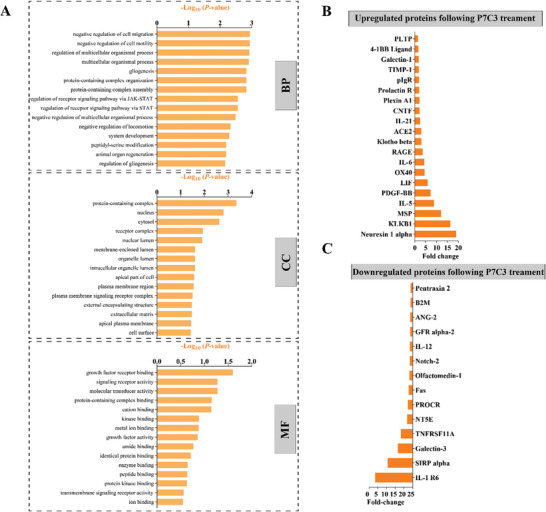
Serum cytokine and chemokine profile following P7C3 treatment versus OVX animals. A] GO enrichment analysis for differentially expressed biomarkers. BP = biological process; CC = cellular component; MF = molecular function. B] The top 20 differentially expressed proteins (upregulated) following P7C3 treatment are presented. C] The top 14 differentially expressed proteins (downregulated) following P7C3 treatment are presented.

In order to investigate the systemic effects of P7C3 on OVX animals, we used a cytokine array to quantify cytokine and chemokine expression in serum, which allowed us to examine 286 rat proteins simultaneously. The proteins were hierarchically clustered, and **Figure** **7**A shows the top 15 gene enrichment (GO) terms in the BP, CC, and MF categories, respectively. The top 5 upregulated GO terms in the BP category were, negative regulation of cell migration, negative regulation of cell motility, regulation of multicellular organismal process, multicellular organismal process and gliogenesis. The top 5 GO terms in the MF categorery included growth factor receptor binding, signaling receptor activity, molecular transducer activity, protein‐containing complex binding, and cation binding. Moreover, the top 5 GO terms in the CC category included protein‐containing complex, nucleus, cytosol, receptor complex, and nuclear lumen. Figure 7B and C shows the top 20 most upregulated proteins and the top 14 most downregulated proteins following P7C3 treatment. Among the highly expressed proteins were neurexin‐1α, kallikrein B1 (KLKB1), MSP, IL‐5, PDGF‐BB, LIF, wheras IL‐1 R6, signal regulatory protein‐α (SIRPα), Galectin‐3, TNFRSF11A (also known as RANK), 5′‐nucleotidase ecto (NT5E), protein C receptor (PROCR), and Fas were among the most downregulated proteins.

**Figure 8:**
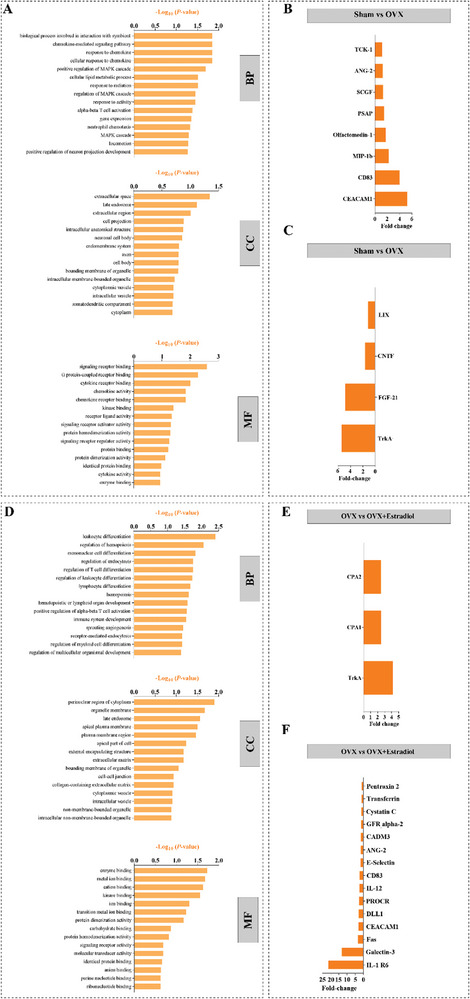
Serum cytokine and chemokine profiles in the Sham versus OVX, and OVX versus OVX + Estradiol groups. A] GO enrichment analysis for differentially expressed biomarkers in Sham versus OVX animals. BP = biological process; CC = cellular component; MF = molecular function. B] The top 8 differentially expressed proteins (upregulated) and, C] The top 4 differentially expressed proteins (downregulated) in Sham versus OVX rats. Similarly, D] GO enrichment analysis, E] the top 3 upregulated, and F] top 15 downregulated proteins in OVX versus OVX + Estradiol animals.

**Figure** **8**A–C shows the top upregulated GO terms in the BP, CC, and MF categories as well as the top up‐ and downregulated serum proteins levels. Most notably, our results show that ovariectomy resulted in a ≈5‐fold increase in CEA cell adhesion molecule 1 (CEACAM1), a ≈4‐fold increase in Cluster of Differentiation 83 (CD83), and a ≈2‐fold increase in MIP‐1β, with a ≈5‐fold reduction in Tropomyosin receptor kinase A (TrkA), and FGF‐21. Ovariectomized rats treated with Estradiol displayed a ≈4‐fold increase in TrkA, and a ≈20‐fold decrease in IL‐1 R6 and a ≈12‐fold reduction in galectin‐3 (Figure 8D–F).

### 2.8 P7C3 Treatment Changes Microbiome Profiles in the Gut

**Figure 9:**
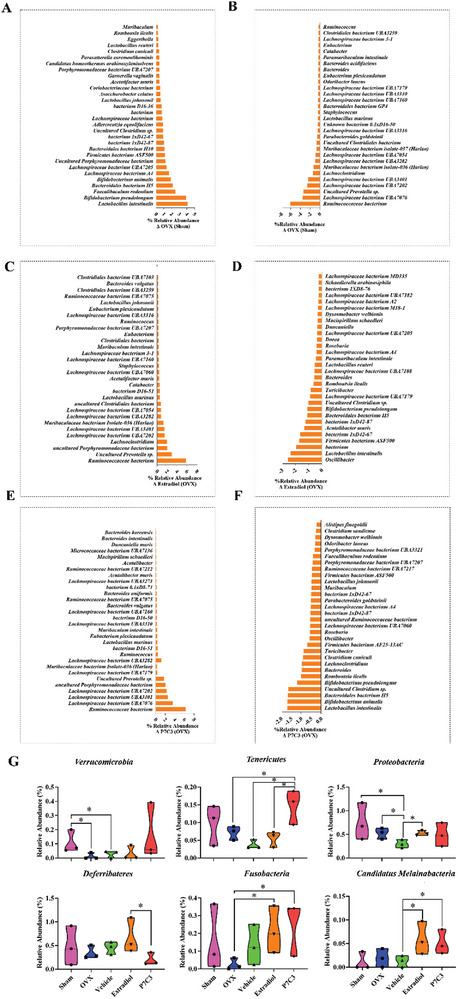
%Relative abundance of bacteria identified within the gut microbiome. A,B] in Sham versus OVX animals, C,D] Sham versus OVX + Estradiol animals, E,F] in Sham versus P7C3 animals. G] While no significant differences in the relative abundance of the major phyla (Firmicutes, Bacteriodetes, Actinobacteria, and unknown) was found between treatment groups, significant differences were observed among several less abundant phyla (Verruomicrobia, Proteobacteria, Tenericutes, Deferribacteres, Fusobacteria and Melainabacteria). Changes in the mean %relative abundance are presented. *p < 0.05.

Investigation of the GM is a novel and rapidly expanding field, where changes in the GM are known to influence skeletal homeostasis as well as osteoporosis.^[^ ^61^, ^79^ ^]^ As such, the GM is emerging as a promising therapeutic target in the management and/or prevention of inflammatory and metabolic disorders.^[^ ^60^ ^]^ Here, we investigated the 16S rDNA abundance of Actinobacteria, Bacteroidetes, Candidatus Melainabacteria, Deferribacteres, Firmicutes, Fusobacteria, Proteobacteria, Tenericutes, and Verrucomicrobia within the GM using Shotgun Metagenomic analyses (Figure S13 and Table S1, Supporting Information). Collectively comprising ≈90% of the human gut microbiome,^[^ ^80^ ^]^ an increased ratio of Bacteroidetes to Firmicutes within the microbiome has been long associated with obesity, elevated low‐grade inflammation,^[^ ^81^ ^]^ and steroid deficiency‐induced osteoporosis,^[^ ^66^ ^]^ despite limited evidence on the directionality of this relationship.^[^ ^60^ ^]^ Our data show healthy sham animals displayed the lowest mean Firmicutes abundance of 57.85 ± 3.62%, with a Bacteroidetes to Firmicutes ratio of 1:3.06. While OVX animals displayed an abundance of 67.63 ± 6.34%, and a ratio of 1:3.18, Estradiol‐treated animals an abundance of 64.78 ± 1.35%, and 1:3.88, P7C3‐treated and abundance of 63.80 ± 3.77%, and a ratio of 1:3.03, while the DMSO‐vehicle group showed an abundance of 66.60 ± 0.44%, and the highest ratio of 1:4.05. While this study did not identify significant differences in the relative abundance of these phyla between treatment groups, significant differences were observed among several less abundant phyla. Notably, ovariectomy induced a significant decrease in the %abundance of Verrucomicrobia when compared to the healthy control animals, while P7C3 treatment significantly increased the %abundance of Tenericutes compared to the OVX, Estradiol and DMSO‐vehicle groups (*p* < 0.05 in all cases, Figure 9G). Further, P7C3 significantly increased the abundance of Fusobacteria compared with OVX animals, significantly decreased Deferribateres when compared with Estradiol‐given animals, while both Estradiol and P7C3 significantly increased Melainabacteria (*p* < 0.05 in all cases).

**Figure 10:**
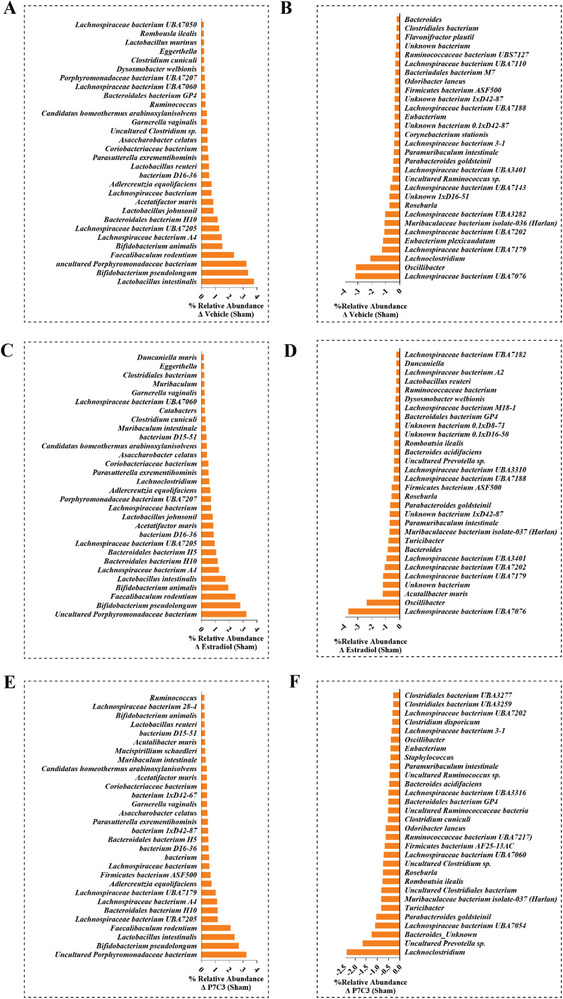
%Relative abundance of bacteria identified within the gut microbiome. A,B] in Sham versus OVX + Vehicle animals, C,D] Sham versus OVX + Estradiol animals, E,F] in Sham versus OVX + P7C3 animals.

At the species level, the top 30 most up‐ and down‐regulated in each group were identified when compared to OVX (**Figure** **9**A–F), and healthy control animals (**Figure** **10**A–F). Figure S14 (Supporting Information) shows the top 30 most up‐ and down‐regulated species following vehicle treatment. Notably, and when compared with control healthy animals, the untreated OVX group *Ruminococcaceae* bacterium was most downregulated (−6.1‐fold), while both Estradiol and P7C3 induced the highest upregulation in *Porphyromonadaceae* bacterium (3.3‐fold). All groups, i.e., OVX control, Estradiol, and P7C3 treated rats, presented with a similar upregulation in the relative abundance of *Bifidobacterium pseudolongum*, *Lactobacillus intestinalis*, and *Faecalibaculum rodentium* (range, ≈2‐4‐fold). In contrast, and when the highest relative abundance levels were assessed in the P7C3 and Estradiol groups versus OVX animals, a substantial 6.24‐fold and 5.85‐fold increase in *Ruminococcaceae* bacterium was measured respectively, together with an increase in several *Lachnospiraceae* bacterium species. Both groups also revealed a decrease in the abundance of *Lactobacillus intestinalis* (−1.69‐fold, and −2.34‐fold respectively).

## 3 Discussion

By 2060, it is projected that one in four Americans will be elderly (≥ 65 years), the number of our oldest‐old (≥ 85 years) will triple, and the country will add 500 000 centenarians.^[^ ^82^ ^]^ Consequently, the prevalence of osteoporosis, the incidence of fragility fractures, and the risk of adverse health outcomes will also undoubtedly increase.^[^ ^83^, ^84^ ^]^ Due to the limitations associated with contemporary medical interventions, the development of novel approaches is necessary to surmount our current and expected medical needs. In this study, we have identified a promising therapeutic approach for mitigating the loss in biomechanical strength, microarchitectural structure, and bone area caused by OVX‐induced osteoporosis using P7C3, an aminopropyl carbazole NAD^+^/NADH stabilizing agent. Importantly, and comparable to the frailty caused by osteoporosis, P7C3 has also shown protective efficacy against additional conditions prevalent in the elderly population. This includes Alzheimer's disease,^[^ ^85^ ^]^ Parkinson's disease,^[^ ^86^, ^87^, ^88^, ^89^ ^]^ peripheral neuropathy,^[^ ^90^ ^]^ and traumatic brain injury,^[^ ^59^, ^91^, ^92^ ^]^ thus indicating P7C3 as a highly promising future therapy able to counter a broad range of age‐related morbidities. These, previous studies have shown that P7C3 is an orally bioavailable compound with a desirable half‐life and volume of distribution, making it a safe option for administering to rodents (intraperitoneal, intravenous, or oral administration^[^ ^50^, ^93^, ^94^ ^]^) even at higher concentrations for extended periods,^[^ ^50^, ^93^ ^]^ including in nonhuman primates over a 38 week period.^[^ ^94^ ^]^ Consistent with previous studies, our results provide further validation for the safety and effectiveness of P7C3 as a therapeutic agent, as we did not observe any histological indications of toxicity in vital organs including the heart, liver, spleen, lung, brain, or kidney up to 13 weeks following treatment.

Despite years of research, the underlying mechanism and pathogenesis of age‐associated osteoporosis remains elusive. Traditionally, osteoporosis has been studied in the context of endocrine dysfunction. Notably, the abrupt decline in estrogen,^[^ ^95^ ^]^ and increase in androgen^[^ ^96^ ^]^ levels following menopause in women, and the gradual decline of both estrogen, and androgen levels in men,^[^ ^4^, ^48^ ^]^ significantly contributes to an imbalance in bone remodeling, leading to bone loss and increased fracture risk. Our data reveals the highest, and >5‐fold reduction in serum levels of TrkA, a neurotrophin expressed by sensory neurons within bone, a central mediator of bone pain,^[^ ^97^ ^]^ a suppressor of osteoprogenitor number,^[^ ^98^ ^]^ and a protein that is essential for bone repair.^[^ ^99^ ^]^ However, its role in osteoporosis remains elusive. Notably, TrkA was the most upregulated protein (≈4‐fold) in Estradiol‐given animals, suggesting the important contribution of this protein in protecting against OVX‐induced bone loss. Further, estrogen delivers an anabolic effect by increasing progenitor recruitment from within the bone marrow niche, in upregulating osteoblastic differentiation, and by reducing osteoblast apoptosis. Estrogen also modulates osteoclastic activity via suppressing the production of RANKL and increasing OPG, as well as coordinating tumor necrosis factor‐alpha (TNFα), IL‐1β, IL‐6, macrophage‐colony stimulating factor, and granulocyte macrophage‐colony stimulating factor; cytokines known to extensively contribute to bone loss.^[^ ^100^ ^]^ This potentially occurs via inhibition of NF‐κB,^[^ ^101^ ^]^ a ubiquitous transcription factor where deregulation of its activity is considered a hallmark of chronic inflammatory diseases,^[^ ^102^ ^]^ and where inhibition promotes bone formation.^[^ ^103^ ^]^ However, more recent studies have discovered that osteoporosis is multifactorial with causes also stemming from immunology, the gut flora, diet, and cellular senescence, among others.^[^ ^104^ ^]^

The primary mechanism for canonical and non‐canonical NF‐κB activation is via stimuli including TNF superfamily members, T‐cell and B‐cell receptors,^[^ ^105^ ^]^ as well as CD40L and RANKL respectively.^[^ ^106^ ^]^ The RANK transmembrane receptors are typically anchored by adaptor proteins such as TNF receptor‐associated factors, which transmit signals to downstream targets including NF‐κB and c‐Jun N‐terminal kinases (JNK).^[^ ^100^, ^107^ ^]^ The RANKL/OPG/RANK/NF‐κB signaling pathway is a key modulator of osteoclast formation and activity, and during the pathogenesis of osteoporosis, chronic inflammation‐induced osteoclastic activity augments bone loss via NF‐κB.^[^ ^108^, ^109^ ^]^ Here, our results reveal that similar to the Estradiol‐treated, positive‐control animals, P7C3 significantly decreased RANKL^+^ and TRAP^+^ expression both in vitro and in vivo. Further, our proteomic analyses show a significant P7C3‐induced −20‐log reduction in the IL‐1 receptor, IL‐1 R6 (also named IL‐1 Rrp2 or IL‐1 RL2), a key activator of NF‐κB,^[^ ^110^ ^]^ via JNK^[^ ^111^ ^]^ and mitogen‐activated protein kinase (MAPK)^[^ ^112^ ^]^ mediated pathways, and following IL‐1 activation.^[^ ^113^ ^]^ Interestingly, IL‐1 R6 is reported to be strongly associated with both the activation of inflammation and chronic inflammatory diseases,^[^ ^111^ ^]^ as well as delivering anti‐inflammatory effects.^[^ ^114^, ^115^, ^116^ ^]^ Most notably, our results reveal P7C3 to be a key regulator of TNFRSF11A (RANK) expression, as evidenced by a significant and >−10‐fold decrease in TNFRSF11A serum protein levels, thus downregulating osteoclastic survival, differentiation, expression of osteoclast‐specific genes,^[^ ^117^, ^118^, ^119^ ^]^ and function via reduced binding with RANKL.^[^ ^120^, ^121^ ^]^ Further, overexpression of RANK alone, is sufficient to activate the NF‐κB pathway.^[^ ^122^ ^]^ Interestingly, RANK‐bearing osteocytes and osteoblasts are often spatially distant from RANK‐containing osteoclasts in vivo. A recent study by Holliday et al.^[^ ^123^ ^]^ highlighted emerging evidence showing both RANK and RANKL as functional components within extracellular vesicles (EVs) released by these cells. Thus, secretion from EVs may facilitate an expansion in the coupling of bone resorption with bone formation at sites distant to the location of each independent cell, further emphasizing the crucial role of RANK and RANKL as drug targets and biomarkers. Nevertheless, over recent years, new approaches aimed to address osteoporosis have largely focused on osteotherapeutics designed to directly target RANKL or OPG, or indirectly effect this signaling pathway via anti‐sclerostin or anti‐cathepsin K approaches, for example.^[^ ^124^, ^125^ ^]^ To the best of our knowledge, this is first evidence of an intervention that drives against bone loss by contributing to a reduction in the expression of RANK.

The janus kinases (JAKs) are a family of protein tyrosine kinases that act on signal transducer and activator of transcription (STAT), a signaling pathway that serves as an important downstream mediator for a variety of cytokines, hormones, and growth factors, as well as for osteoblast, and osteoclast proliferation and differentiation.^[^ ^126^, ^127^ ^]^ Further, emerging evidence suggests STAT3 and its network, participates in the development of osteoporosis.^[^ ^128^ ^]^ Our GO data reveal the significant P7C3‐induced increase in regulation of receptor signaling pathway via JAK/STAT and STAT. To support this, Adam et al.^[^ ^129^ ^]^ reported that JAK/STAT inhibition stimulated osteoblast function, increased bone mass, reduced osteoclast activity, thereby preventing OVX‐induced bone loss via RANKL/NF‐κB signaling in a murine model. Similarly, Li et al.^[^ ^130^ ^]^ showed that JAK inhibition prevented RANKL‐mediated osteoclastogenesis, and prevented OVX‐induced osteoporosis by suppressing activation of STAT3 and NF‐κB pathways. Finally, Fu et al.^[^ ^131^ ^]^ and using microRNA, demonstrated that upregulation of JAK/STAT signaling reduced the progression of OVX‐induced osteoporosis. Our data suggests a pivotal characteristic of P7C3 anti‐osteoporotic activity may be in regulating both JAK/STAT and NF‐κB signaling.

Our results further reveal specific P7C3‐induced upregulation in the tyrosine kinase receptor activities of FGFR3, IGF‐1R, Ryk, and EphB2, cell‐to‐cell mediators who all play critical roles in bone homeostasis. Notably, FGFR3 activates the NF‐κB^[^ ^132^ ^]^ and STAT^[^ ^133^ ^]^ signaling pathways, and reportedly directly promotes or inhibits the osteogenic differentiation and mineralization of BMSCs.^[^ ^134^, ^135^ ^]^ Further, FGFR3 knockout mice display decreased bone mass via disruption of both osteoblastogenesis and osteoclastogenesis.^[^ ^135^ ^]^ The IGF‐1R signaling pathway is critical for bone cell proliferation and differentiation, and maintaining bone mass,^[^ ^136^ ^]^ via regulation of caspase‐3 protease, a molecule central to signal osteoblasts to deposit bone and in NF‐κB signaling.^[^ ^136^, ^137^, ^138^, ^139^ ^]^ Further, studies have determined relationships between deficiencies in the IGF system and increased rates of fracture in both men and women.^[^ ^140^, ^141^ ^]^ Ryk is an atypical Wnt receptor predominantly expressed by BMSCs, and able to support the self‐renewing expansion of primitive hematopoietic progenitors, thereby modulating the bone marrow niche response to canonical Wnt ligands.^[^ ^142^ ^]^ Finally, the erythropoietin‐producing human hepatocellular (Eph) receptor family are expressed by stromal, hematopoietic, and vascular populations and mediate contact‐dependent cross‐talk within the bone microenvironment,^[^ ^143^, ^144^ ^]^ and promote mineral formation.^[^ ^145^, ^146^ ^]^ Specifically, EphB2 has been shown to negatively regulate osteoclast differentiation and function, inhibit TRAP^+^ cell expression, resorption activity, the expression of RANK, and Cathepsin K among others.^[^ ^147^, ^148^, ^149^ ^]^ EphB2 is responsive to biomechanical load in mice,^[^ ^150^ ^]^ and EphB2 osteoblast deletion increased apoptosis in vitro, and significantly reduced bone formation in knockout mice in vivo.^[^ ^151^ ^]^ Together, these results show P7C3 targets multiple key molecules able to modulate bone formation and loss via RANK/RANKL/OPG/NF‐κB, JAK/STAT signaling and by modulating the intensity of Wnt/β‐catenin signaling.

After administering P7C3, our serum protein array analyses also identified significant upregulation in several important cytokines, including MSP, IL‐5, PDGF‐BB, LIF. Macrophage‐stimulating protein has been reported to enhance osteoblastic differentiation via the extracellular signal‐regulated kinase (ERK) signaling pathway,^[^ ^152^ ^]^ while also increasing osteoclast survival, but not their activation.^[^ ^153^ ^]^ IL‐5 overexpression mediates bone formation through the mobilization of marrow‐derived osteogenic progenitors, while also inhibiting the recruitment of osteoclasts.^[^ ^154^ ^]^ Platelet‐derived growth factor‐BB is expressed on preosteoclasts^[^ ^155^ ^]^ and considered to play an important regulatory role in augmenting the proliferation of BMSCs and their osteogenic differentiation in vitro,^[^ ^156^, ^157^ ^]^ promoting angiogenesis,^[^ ^158^ ^]^ and in bone tissue repair and regeneration.^[^ ^159^, ^160^, ^161^ ^]^ Further, an in vivo rat model showed PDGF‐BB secreted by preosteoclasts was pivotal to coupling angiogenesis with osteogenesis, where low PDGF‐BB levels in serum and bone marrow were reported in an OVX‐induced osteoporotic mouse model.^[^ ^155^ ^]^ A study by Tang et al. reported postmenopausal women, especially those with postmenopausal osteoporosis, displayed significantly lower plasma Estradiol and PDGF‐BB levels compared to “normal” healthy young women.^[^ ^162^ ^]^ Interestingly, and in an OVX rat model, estrogen maintained PDGF‐BB levels suggesting alterations in PDGF‐BB plays a significant role in postmenopausal osteoporosis. LIF is a multifunctional cytokine belonging to the IL‐6 family,^[^ ^163^, ^164^ ^]^ that has a positive effect on osteoblast differentiation, chondrocyte, osteocyte, and adipocyte activity,^[^ ^164^ ^]^ bone formation and regeneration,^[^ ^165^, ^166^ ^]^ maintenance of hematopoietic stem cells,^[^ ^167^ ^]^ and angiogenesis.^[^ ^168^ ^]^ In addition, LIF secreted by osteoclasts,^[^ ^169^, ^170^ ^]^ downregulates sclerostin expression, a glycoprotein mainly secreted by osteocytes, which functions to upregulate bone formation via the Wnt/β‐catenin signaling pathway in osteoblasts.^[^ ^171^, ^172^ ^]^ Interestingly, sclerostin has also been reported to enhance adipocyte‐specific gene expression, and the accumulation of lipid deposits,^[^ ^173^ ^]^ and adipogenesis,^[^ ^174^ ^]^ and body fat composition,^[^ ^175^ ^]^ serving as a key regulator of both bone and fat metabolism in part, through Wnt/β‐catenin signaling. Thus, via increased LIF, which in turn downregulates osteocyte‐released sclerostin, P7C3 may have upregulated bone and downregulated fat tissue formation, contributing to the reduction in adipose tissue measured in P7C3‐treated rats when compared to the OVX, and OVX DMSO vehicle‐control groups.

The accumulation of bone marrow adiposity is a common occurrence in aging individuals and those with certain pathological conditions, such as ionizing radiation exposure,^[^ ^176^ ^]^ diabetes mellitus,^[^ ^177^ ^]^ and post‐menopausal osteoporosis.^[^ ^178^, ^179^ ^]^ Age‐related changes in the functional capacity of MSCs, including impaired proliferation and a switch in lineage commitment toward adipogenic differentiation at the expense of osteoblastogenesis, have been identified as major contributors to the development of osteoporosis.^[^ ^180^ ^]^ Our in vitro studies show that P7C3 treatment significantly reversed BMSC adipogenic differentiation by inhibiting adipogenic transcription factors, such as SREBP‐1, and lipid droplet formation, while promoting osteoblastogenesis, as evidenced by a significant increase in ALP expression, and bone mineral deposition via upregulation of c‐Fos, c‐Jun, TAK1, SMAD‐1, −2, and −5. This suggests the osteogenic differentiation, proliferation, and maturation observed in vitro, occurred via c‐Fos and c‐Jun heterodimerization, as well as by positive upregulation of SMAD‐1, −2 and −5, proteins associated with the well‐established canonical TGFβ signaling pathway, a vital regulator of bone formation via both osteoblastic and osteoclastic activity.^[^ ^181^ ^]^ Our proteomic analyses further support P7C3‐induced TGFβ signaling via the SMAD pathway as indicated by the upregulation of BMP6 and BMP7. BMP6 and BMP7 bind to various TGFβ receptors, leading to the recruitment and activation of SMAD family transcription factors, thereby regulating bone and fat tissue development.^[^ ^182^ ^]^ Further, PDGF‐BB was also upregulated and is a top upstream regulator for TGFβ signaling via the SMAD pathway.^[^ ^183^ ^]^ Notably, DKK‐1 an antagonist of the Wnt/β‐catenin signaling pathway, was also upregulated. Together these data suggest that P7C3‐induced osteogenic differentiation occurred in part, by both TGFβ and Wnt/β‐catenin signaling activity. In support of this, our in vitro proteomic analyses obtained from osteoclast lysate further support the critical role of P7C3‐induced TGFβ signaling in promoting osteogenesis. Data reveal major upregulation of VE‐Cadherin, a critical regulator of TGFβ signaling,^[^ ^184^ ^]^ as well as major downregulation of endoglin, a protein also known to modulate TGFβ signaling,^[^ ^185^ ^]^ where decreased expression correlates with increased osteogenesis.^[^ ^186^ ^]^ GDF11 was substantially downregulated and is also a member of the TGFβ superfamily. GDF11 suppresses expression of the osteogenic protein Runx2 and osteoblast differentiation, as well as stimulates RANKL‐induced osteoclastogenesis by inducing SMAD2/3 phosphorylation.^[^ ^187^, ^188^ ^]^ Further, decreased levels of IL‐6‐like protein, CNTF, were noted where increased expression is reported to inhibit mineralization and trabecular bone formation,^[^ ^189^ ^]^ and its secretion by osteoclasts may play a role in the coupling process by which osteoclasts recruit osteoblasts.^[^ ^190^ ^]^ Favorable osteoblast over osteoclastic activity was also evident by downregulation of the protein CTGF, which facilitates the cell‐to‐cell fusion of osteoclast precursors, and multinucleation and maturation of osteoclasts.^[^ ^191^ ^]^ Finally, increased Fas levels were measured, and an elevation is Fas is associated with the induction of osteoclast apoptosis.^[^ ^192^, ^193^ ^]^ Further, our in vivo OVX model reveals similar to Estradiol, that P7C3 offers a systemic protective outcome, as it effectively reduced the abnormal accumulation of not only bone marrow adiposity, but also subcutaneous, and visceral fat, thereby vitiating the significant whole‐body weight gain observed in both the OVX, and OVX DMSO‐vehicle control groups. Here, our data shows that in the untreated ovariectomized control group, OVX induced a ≈5‐fold reduction in FGF21 protein serum levels. Mechanistically, FGF21, which is primarily produced by adipose tissue,^[^ ^194^ ^]^ inhibits osteoblastogensis and stimulates adipogenesis from BMSCs via PPAR‐γ,^[^ ^195^ ^]^ and has been shown to have a positive clinical relationship with osteoporosis.^[^ ^196^ ^]^ The expression of FGF21 in this group, along with a ≈5‐fold increase in CEACAM1 expression, is likely significantly associated with the increase in bone marrow adiposity and whole‐body weight gain measured in this group.^[^ ^197^ ^]^ Estrogen has been shown to reduce adipogenesis through the direct inhibition of PPARγ,^[^ ^198^, ^199^, ^200^, ^201^ ^]^ however, our in vitro investigations reveal no P7C3‐induced reduction in this major adipogenic regulatory gene, suggesting the induction of an alternative adipogenic route. Insulin receptor was also upregulated by P7C3. Insulin receptor signaling, (via the insulin/IGF‐1R signaling pathway), controls osteoblast development and osteocalcin expression, where knockout mice displayed decreased bone formation due to a deficient number of osteoblasts.^[^ ^202^ ^]^ Notably, and with age, these mice develop marked adiposity and hyperglycemia, indicating a bone‐endocrine (osteocalcin) loop that regulates bone and fat tissue formation via glucose metabolism. To this end, Clemens and Karsenty^[^ ^203^ ^]^ highlighted the importance of the osteoblast as a target used by insulin to control whole‐body glucose homeostasis, and identified bone resorption as the mechanism regulating osteocalcin activation. Additionally, lipid accumulation in adipocytes activates c‐Jun N‐terminal (JNK) and NF‐κB pathways,^[^ ^204^ ^]^ which were both potentially downregulated by P7C3, and this may have contributed to the reduced level of adipogenesis seen. Finally, our data showed that both Estradiol, and P7C3 treatment significantly downregulated serum levels of galectin‐3. Takada et al.^[^ ^205^ ^]^ demonstrated that a high level of galectin‐3 was detected in obesity‐associated intermuscular adipose tissue, and this protein was found to activate adipogenic PPARγ signals in PDGFRα^+^ cells. Maupin and colleagues^[^ ^206^ ^]^ discovered that galectin‐3 functions as a negative regulator of bone formation and that female mice lacking galectin‐3 were protected from age‐related trabecular bone loss. Paradoxically, Iacobini et al.^[^ ^207^ ^]^ demonstrated that galectin‐3 knock out mice exhibit accelerated age‐dependent trabecular bone loss compared to wild‐type mice. Therefore, further research is warranted to investigate the role of P7C3 in regulating LIF, insulin receptor, and galectin‐3, and the subsequent response to bone and fat metabolism. Finally, both Estradiol and P7C3 downregulated levels of the protein Fas, where increased Fas expression has been shown to contribute to adipose tissue dysfunction, mediate inflammatory signals in obesity, and reduce adipogenesis.^[^ ^208^, ^209^, ^210^ ^]^ Reduced levels of Fas are also associated with an increased resistance to OVX‐induced bone loss via increase OPG expression.^[^ ^211^ ^]^

Changes within the gut microbiota also contribute to alterations in fat and bone metabolism. At the species level, *Porphyromonadaceae* bacterium is reported to be negatively correlated with glucose and lipid metabolism, and weight gain in mice,^[^ ^212^ ^]^ play a pivotal role in murine host‐lipid metabolism,^[^ ^213^ ^]^ with elevated levels associated with reduced visceral adipose tissue and a healthier metabolic profile in elderly individuals.^[^ ^214^ ^]^ Further, *Porphyromonadaceae* bacterium plays a key role in eliciting an anti‐inflammatory response by inhibiting TNFα, IL‐1β and IFNγ.^[^ ^215^, ^216^ ^]^ Notably, the most upregulated species (≈3.3‐fold) within the GM in both P7C3 and Estradiol given animals was *Porphyromonadaceae* bacterium. While Candidatus Melainabacteria (Cyanobacteria) comprised <0.1% of microbiota, both Estradiol and P7C3 displayed significantly high abundance compared to all other groups. Notably, low levels of Melainabacteria has been associated with obesity,^[^ ^63^ ^]^ but this area remains in its infancy. In contrast, and following ovariectomy, and in both the untreated and Estradiol‐given group, data showed a variety of 10 and 9 different *Lachnospiraceae spp*. respectively, were downregulated. However, in P7C3‐treated animals when compared to healthy control animals, a total of 5 *Lachnospiraceae spp*. were downregulated, and to a much lesser amount. In comparison, P7C3‐treated animals versus OVX‐only animals showed the increased abundance of a total of 8 *Lachnospiraceae spp*. When in high abundance, a positive correlation between *Lachnospiraceae spp*. and glucose and/or lipid metabolism, leading to obesity has been reported.^[^ ^217^, ^218^ ^]^ However, Ni et al.^[^ ^219^ ^]^ recently reported that Lachnospiraceae are positively correlated with bone formation and mass. As such, its role/s within the GM is considered complex and is currently controversial.^[^ ^220^ ^]^ Finally, *Ruminococcaceae* bacterium (assigned to the Lachnospiraceae family) is considered an important contributor to intestinal health, and healthy metabolic and immune function.^[^ ^221^ ^]^ Low levels have been reported to be associated with the elderly, and frailty,^[^ ^64^, ^65^ ^]^ and positively correlated with osteoclastic indicators and bone loss in OVX rats.^[^ ^66^ ^]^ Our results show that ovariectomy induced the highest downregulation (≈−6‐fold) in Ruminococcaceae bacterium,^[^ ^222^ ^]^ when compared with Estradiol and P7C3 treated animals. Notably, *Lactobacillus*, and *Bifidobacterium* were upregulated in the OVX, DMSO‐vehicle, P7C3, and estradiol groups when compared to control animals, while substantially downregulated in the P7C3 versus OVX group. Both families have been shown to positively regulate the systemic level of leptin,^[^ ^62^ ^]^ and with varying roles in augmenting bone metabolism in humans and rodents.^[^ ^218^, ^223^, ^224^, ^225^, ^226^ ^]^ These may have also directly affected the adipo‐osteogenic interactions, and inflammatory cytokine (e.g., TNFα and IL‐1β^[^ ^227^, ^228^ ^]^) response observed in this study.

NAD^+^‐mediated oxidative phosphorylation has been shown to restore mitochondrial fitness and cellular homeostasis in senescent BMSCs,^[^ ^229^ ^]^ and is indispensable for osteoblastogenesis and bone regeneration, where attenuates of NAD^+^ impair the osteogenic differentiation of BMSCs in vitro and diminish fracture healing in vivo.^[^ ^230^ ^]^ Notably, restoration of the reduced NAD^+^ levels measured in age‐related osteoporosis,^[^ ^55^ ^]^ decelerated bone loss with enhanced osteogenesis and suppressed adipogenesis via a Sirt1‐dependent pathway.^[^ ^231^, ^232^ ^]^ Further, increased NAD^+^/NADH has been shown to reduce proinflammatory activation,^[^ ^233^, ^234^, ^235^ ^]^ while the gut microbiome plays a role in beneficially modulating NAD^+^ bioavailability in vivo,^[^ ^236^ ^]^ through boosting mammalian host NAD^+^ metabolism.^[^ ^237^ ^]^ As such, and theoretically, there is a close correlation between NAD^+^, gut microbiota, and host metabolism^[^ ^238^ ^]^ however their interactions remain largely unexplored. These studies support the NAD^+^/NADH‐stabilizing properties of P7C3^[^ ^49^, ^50^ ^]^ together with the results revealed in this study, and suggest the osteo‐adipogenic lineage fate, and senescent state of BMSCs, proinflammatory response, and alterations in gut microbiota are all influenced by cell energy metabolism. Thereby, data indicates an important NAD^+^ dependent role in the development of osteoporosis.

Finally, our results also show significant upregulation of the key neurogenic factor neurexin‐1α, which influences synapse function and neuronal connectivity,^[^ ^239^, ^240^ ^]^ with major downregulation of SIRPα, expressed on myeloid cells and neurons.^[^ ^241^, ^242^ ^]^ Further, KLKB1, a blood coagulation factor,^[^ ^243^ ^]^ was also significantly upregulated in P7C3 treated animals. Although their effect, in addition to the neurotrophin, TrkA is evidently significant in this study, it remains unclear how osteoneuronal activity contributed to the protection in bone loss, and maintenance in bone strength observed following OVX. As such, further investigation is highly warranted. While this study focused on the anti‐osteoporotic role of P7C3 in OVX animals, a limitation was the exclusion of a P7C3 treated sham control group, and as such, this requires further investigation. Our in vivo analyses demonstrated that despite ovariectomy, bone area, and strength were maintained following the administration of P7C3. However, notably, differences in bone turnover rates were not investigated, and as such, new bone formation was not assessed in this study. Further, increased estrogen levels have been associated with decreased eating,^[^ ^244^ ^]^ and an additional limitation to this study is that food and water uptake were not quantified.

## 4 Conclusion

**Figure 11:**
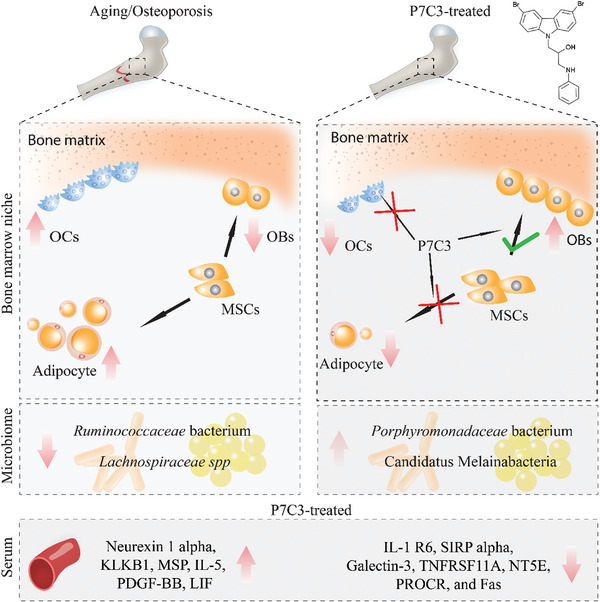
Schematic diagram showing the protective effect provided by exogenous administration of P7C3 against OVX‐induced osteoporosis. The process of aging, particularly in post‐menopausal women who experience estrogen deficiency, can lead to weaker bones that are more susceptible to fractures. This condition is characterized by a loss of bone mass, increased osteoclast activity, an accumulation of marrow adiposity, and adipogenic weight gain. Here, and to the best of our knowledge, we are first to discover that exogenous administration of 20 mg k^−1^g P7C3 can shift the pathological environment induced by osteoporosis from favoring osteoclastogenesis and adipogenesis into osteogenesis, thereby providing significant protection to bone against osteoporosis‐mediated bone loss and fracture in vivo. P7C3‐induced alterations within the gut microbiome may also contribute to the favorable result observed.

In conclusion, our study shows that P7C3 treatment inhibits osteoclast maturation, and osteoclastic activity, and can shift the fate of hBMSCs toward osteogenic differentiation while inhibiting adipogenesis in vitro. The administration of P7C3 to OVX rats in vivo resulted in the preservation of bone area, architecture, and mechanical strength, while significantly suppressing RANK, RANKL, osteoclastogenesis, bone marrow, and whole‐body adipogenesis (**Figure** **11**). To the best of our knowledge, this is first evidence of an intervention that drives against bone loss by contributing to a reduction in the expression of RANK. Our data also suggests a pivotal characteristic of P7C3 anti‐osteoporotic activity is in modulating adipo‐osteogenic lineage commitment, via the TGFβ/SMAD, and Wnt/β‐catenin signaling pathways, and potentially via the regulation of JAK/STAT, and NF‐κB signaling. As such, further focused and mechanistic analyses are warranted to investigate the contribution of RANK, JAK/STAT, and NF‐κB signaling in the P7C3‐mediated therapeutic response observed. Further, P7C3 increased the relative abundance of *Porphyromonadaceae* bacterium, and Candidatus Melainabacteria, while reducing *Ruminococcaceae* bacterium, which may also have beneficially contributed to favorable regulation of inflammation, bone, and adipose metabolism. Future analyses are necessary to determine the anti‐osteoporotic effect and the potential synergistic contribution that P7C3 confers to directly regulating both bone tissue and the gut microbiome. Together, our results reveal that P7C3 has the potential to serve as a promising, novel, and versatile therapeutic strategy to promote bone regeneration and enhance fracture resistance in postmenopausal, and potentially other forms of bone loss, by targeting several crucial pathways associated with osteoporosis.

## 5 Experimental Section

### X‐Ray Photoelectron Spectroscopy

The chemical composition of P7C3 was analyzed using X‐ray photoelectron spectroscopy (XPS). The powdered form of P7C3 was loaded onto a sample holder and the XPS spectrum was recorded using an ESCALAB‐250Xi spectrometer at room temperature. The X‐ray used for the analysis was generated from a monochromatic Al‐Kα radiation source operating under a vacuum condition of 7 × 10^−9^ mbar and a power of 300 W (15 kV, 20 mA). The X‐ray beam's spot size was fixed at 200 µm, and the C1s peak at 284.6 eV served as a reference peak for peak shift correction. Advantage software was employed to analyze the XPS spectrum and identify the elements present.

### In Vitro Analyses

*Cell culture and reagents*: Human bone marrow‐derived mesenchymal stromal cells (hBMSCs, ATCC PCS‐500‐012) were cultured in complete cell culture medium containing Dulbecco's Modified Eagle's Medium (DMEM; Thermo Fisher Scientific, USA) supplemented with 10% fetal bovine serum (FBS; Thermo Fisher Scientific, USA), and 1% (v/v) penicillin/streptomycin at 37 °C in a humidified incubator with CO_2._ P7C3 (500 mg, HY‐15976) was obtained from MCE (MedChem Express, USA) and was dissolved in dimethyl sulfoxide (DMSO; Fisher Scientific, USA) at a stock concentration of 10 mM for in vitro analysis.

### Analyses of P7C3 on hBMSC Metabolic Activity and Morphology

To assess the effects of P7C3 on hBMSCs' metabolic activity and morphology, an MTT assay was conducted following this previously described protocol.^[^ ^176^ ^]^ Briefly, hBMSCs were exposed to P7C3 at a concentration of either 1 or 10 µM for 3 and 5 days. 1 or 10 µM of DMSO were used as vehicle controls. The absorbance was measured at 570 nm using a microplate reader (Agilent BioTek Synergy H1 Hybrid Multi‐Mode Reader, USA). Additionally, changes in cell number and morphology were evaluated using confocal laser scanning microscopy. Briefly, the cells were fixed with 4% paraformaldehyde for 20 min and stained using Alexa Fluor 594 Phalloidin (A12381, Thermo Fisher Scientific, USA) to visualize the actin filaments, and 4′,6‐Diamidino‐2‐phenylindole dihydrochloride (DAPI; D9542, Millipore Sigma, USA) to identify the nuclei. Images were taken using a confocal laser scanning microscope (Zeiss, USA).

### Osteogenic Differentiation of hBMSCs

To induce the osteogenic differentiation of hBMSCs, complete cell culture medium was supplemented with osteogenic components, including 2 mM β‐glycerophosphate, 100 µM L‐ascorbic acid 2‐phosphate, and 10 nM dexamethasone. The cell culture media was replenished every three days, and contained either 1 or 10 µM P7C3, or the DMSO vehicle control.

### Alkaline Phosphatase Staining

Alkaline phosphatase (ALP) expression was evaluated using an ALP Kit (K2035‐50, BioVision, USA), and according to the manufacturer's instructions. After 7 days of osteogenic hBMSC differentiation with or without P7C3 treatment, cells were stained with ALP solution for 30 min resulting in the purple staining of ALP‐positive cells. Images were acquired using an inverted phase microscope (BZ‐X800E, Keyence, USA).

### Alizarin Red S Staining

To assess the mineralization capacity of hBMSCs at 14 and 28 days, the Alizarin red S (ARS) staining assay was performed. In brief, the cells were fixed with a 4% paraformaldehyde solution for 20 min at room temperature and then incubated with a 2% solution of alizarin red S (A5533‐25G, Millipore Sigma, USA) at a pH 4.1, for 20 min at room temperature. After washing 5 times with deionized water, images were captured using an inverted phase microscope (BZ‐X800E, Keyence, USA). The mineral deposition was dissolved with 10% cetylpyridinium chloride (C0732‐100G, Sigma, USA), and the absorbance was measured using a SpectraMax iD5 Multi‐Mode Microplate Readers (USA).

### Human Bone Metabolism Array

Following the manufacturer's instructions, a human bone metabolism array analysis (AAH‐BMA‐2‐2, Raybiotech, USA) was performed. Following 21 days of osteogenic differentiation, cell samples were harvested for analysis. Protein quantification was conducted using the Pierce BCA Protein Assay Kit (23227, Thermo Fisher Scientific, USA) prior to array analysis. Equal amounts of protein were used for all array analyses.

### Human TGFβ Pathway Phosphorylation Array

The human bone metabolism array (AAH‐TGFB‐1‐2, Raybiotech, USA) was performed following the manufacturer's instructions. Briefly, hBMSCs were either exposed to 10 µM P7C3 or 10 µM DMSO for 1 h in osteogenic differentiation medium. Cell samples were harvested for array analysis. Protein quantification was conducted using the Pierce BCA Protein Assay Kit (23227, Thermo Fisher Scientific, USA) prior to array analysis. Equal amounts of protein were used for all array analyses.

### Adipogenic Differentiation of hBMSCs

To induce adipogenic differentiation in hBMSCs, the cells were cultured using the MesenCult Adipogenic Differentiation Kit (05412, STEMCELL Technologies, USA), which is specifically formulated for the in vitro differentiation of hBMSCs into adipogenic lineage cells. The cell culture media was replaced every 3 days and contained either 1 or 10 µM P7C3, or DMSO vehicle control.

### Lipid Droplet Staining

To detect intracellular lipid droplet formation at 14 and 21 days, a LipidSpot Lipid Droplet Staining Kit (70065, Biotum Inc., Fremount CA, USA) was used per the manufacturer's protocol. In brief, cells were fixed with 4% paraformaldehyde at room temperature for 10 min and then stained with LipidSpot Lipid Droplet Staining Solution for 30 min. Images were captured using an inverted phase microscope (BZ‐X800E, Keyence, USA).

### Quantitative Real‐Time Reverse‐Transcription Polymerase Chain Reaction (qRT‐PCR)

qRT‐PCR was used to measure the expression levels of adipogenesis‐related genes in hBMSCs at 12 days. Total RNA was extracted from the cells using a PureLink RNA Mini Kit (12183018A, Thermo Fisher Scientific, USA) and treated with DNase (PureLink DNase Set, 12185010, Thermo Fisher Scientific, USA) to remove genomic DNA contamination. The RNA concentration was quantified using a NanoDrop 8000 spectrophotometer (NanoDrop Technologies), and cDNA was synthesized using the SuperScript VILO cDNA Synthesis Kit (11756050, Thermo Fisher Scientific, USA) with 500 ng of total RNA as a template. qRT‐PCR was performed using Fast SYBR Green Master Mix (4385612, Thermo Fisher Scientific, USA) on an ABI Prism 7500 Thermal Cycler (Applied Biosystems, Foster City, California, USA) with KiCqStart Primers purchased from Millipore Sigma. The fold change of relative mRNA expression was calculated using the comparative Ct (2^−ΔΔCT^) method.

### Osteoclast Differentiation of Human Osteoclast Precursor Cells

Human osteoclast precursor cells (2T‐110, Lonza, USA) were purchased from Lonza and cultured according to the manufacturer's protocol. Briefly, the cells were cultured in OCP– Osteoclast Precursor Basal Medium containing 10% FBS, 2 mM L‐glutamine, 1% (v/v) penicillin/streptomycin, and 33 ng mL^−1^ M‐CSF (Peprotech) in a humidified incubator containing 5% CO_2_ at 37 °C. To generate mature osteoclasts, the cells were cultured with 66 ng mL^−1^ RANKL (R&D Systems, USA). The live/dead assay was performed according to the protocol developed by ibidi, USA. After staining, images of live (green) and dead (red) cells were taken using a confocal microscope (BZ‐X800E, Keyence, USA). The mature osteoclasts were validated using Tartrate‐resistant acid phosphatase (TRAP) staining, according to our previously published protocol.^[^ ^176^ ^]^ TRAP‐positive multinucleated cells with three or more nuclei were considered as mature osteoclasts.^[^ ^245^ ^]^

### Human L507 Array

The supernatant obtained from various groups following osteoclastic differentiation were collected for the Human L507 Array (AAH‐BLG‐1‐4 RayBiotech Life, GA, USA) accordingly to the manufacturer's protocol. All analyses were conducted in the R programming language V4.2.3 (R Core Team 2017).

### In Vivo Analyses

*Experimental design and P7C3 treatment*: Thirty healthy female SAS Sprague Dawley rats 8–9 weeks of age (≈200 g) were purchased from Charles River. Twenty‐four had undergone ovariectomy (OVX) prior to purchase, and the non‐OVX rats in the control group were appropriately age matched. Rats were allowed to acclimate for one week before starting the study. All animal procedures were approved by the Institutional Animal Care and Use Committee at the University of Central Florida (protocol 2020–48) and were carried out following the guidelines of the American Veterinary Medical Association. The rats were randomly assigned into one of five experimental groups, and each consisting of six animals (*n* = 6): i) sham control (Sham), ii) OVX‐operated (OVX), iii) OVX‐operated + DMSO vehicle control (vehicle), iv) OVX‐operated + 17β‐Estradiol (Estradiol), and v) OVX‐operated + P7C3 at a dose of 20 mg k^−1^g (P7C3). Estradiol and P7C3 injections were performed daily via intraperitoneal (*i.p*.) injection (4 times/week). Differences in body weight were recorded weekly. In vivo live imaging was acquired in rats using the Xtreme II In Vivo imager (Bruker, Billerica, MA). During this time, the rats were maintained on a 12:12‐h light‐dark schedule and given ad libitum access to a standard rodent diet (D07020902, Research Diets, Inc, New Brunswick, NJ, USA) and water. Animals were euthanized 13 weeks following initiation of treatment (≈16 weeks post‐OVX).

### Assessment of Bone Strength via 3‐Point Bending

Immediately after dissection, the retrieved tibiae were wrapped in plastic and stored at ‐20 °C until analysis (*n* = 6). The thawed samples were subjected to 3‐point bending analyses within 96 h of tissue retrieval. Each tibia was placed horizontally on support bars positioned 8 mm apart with the anterior bow facing downward as per our previously published protocol.^[^ ^246^, ^247^ ^]^ Using a universal testing machine (Criterion 43, MTS, Eden Prairie, MN, USA), a vertical force was applied to the tibial mid‐shaft using a 3 mm diameter leading roller and a 5 kN load cell. Each tibia was loaded until failure at a displacement rate of 0.02 mm^−1^s. As the cross‐sectional area of the tibia was non‐uniform and similar to other studies,^[^ ^246^, ^248^, ^249^ ^]^ we assumed the cross‐sectional area was circular and obtained the mechanical properties including the stress, σ (Pa), in Equation (1), and elastic modulus, *E* (Pa), in Equation (2).

(1) $$\sigma \;=\;\frac{F*L*c_{O}}{4*I}$$

(2) $$E\;=\;\frac{FL^{3}}{d*48*I}$$

Where *F* is the applied load (N), *L* = 0.008 is the span distance between the supports (m), *c_o_* is the outer radius of the tibial midshaft (m), which was measured using a caliper (Digital, Cole‐Parmer, IL, US), *d* is displacement (m), and *I* is the moment of inertia (m^4^) calculated using Equation (3):

(3) $$I\;=\;\frac{\pi }{4\left(c_{o}^{4}-c_{i}^{4}\right)}$$

where *c_i_* is the inner radius of the tibial midshaft (m). The inner radius of the midshaft was measured using the µCT scans. Four cross‐sections per bone and from each experimental group were chosen, and the inner and outer diameters were measured for each section. The mean inner‐to‐outer diameter value was used to determine the inner radius. Ultimate stress (σu), yield stress (σy), and fracture stress (σf) were then computed. The mechanical strength parameters were normalized for body size using the ratio of body weight to tibial length.^[^ ^250^ ^]^

### Whole‐Mount Histological Staining for Bony Structure and Major Organs

To perform histological analysis, the bones were extracted, cleaned to remove soft tissue, fixed in 10% neutral buffered formalin, and decalcified in 10% EDTA solution. The processed bones were then embedded in paraffin wax and longitudinal sections were prepared through the distal femoral condyle. 5 µm thick sections were obtained from the paraffin‐embedded samples, and haematoxylin and eosin (H&E) staining was performed using a kit (ab245880, Abcam, USA) according to the manufacturer's instructions to detect the cellular bony structure. Images of H&E‐stained sections were captured using an inverted phase microscope (BZ‐X800E, Keyence, USA).

For assessment of the major organs, including the heart, liver, spleen, lung, kidney, and brain, they were harvested from euthanized rats and fixed in 10% neutral buffered formalin. The organs were then paraffin‐embedded, and 5 µm thick sections were obtained for histological analysis. H&E staining was performed as described above. Images of the stained sections were captured using an inverted phase microscope (BZ‐X800E, Keyence, USA).

### TRAP Staining for Osteoclastic Activity

TRAP staining was performed to evaluate osteoclastic activity, following our previously described protocol.^[^ ^176^ ^]^ Briefly, 5 µm paraffin sections were deparaffinized and rehydrated before being immersed in TRAP staining solution I, consisting of 0.2 M sodium acetate buffer (S2889‐250G, Millipore Sigma, USA) and 50 mM L‐(+) Tartaric acid (228729‐100G, Millipore Sigma, USA) for 20 min at room temperature. Subsequently, an equal volume of TRAP staining solution II was added, which contained 0.5 mg mL^−1^ naphtol AS‐MX phosphate (N4875‐100MG, Millipore Sigma, USA) and 1.1 mg mL^−1^ Fast Red TR salt (ab146351, Abcam, USA), and the solution incubated for 3 h at 37 °C. After washing with distilled water, the cell nuclei were counterstained with hematoxylin solution for 5 mins before mounting with VectaMount AQ Mounting Medium (Vector Laboratories, USA). Stained samples were visualized using an inverted phase microscope (BZ‐X800E).

### ELISA

The ELISA assay for the bone resorption marker, C‐terminal end of the telopeptide of type I collagen (CTX‐1), was performed using rat sera, and according to the manufacturer's instructions (NBP2‐69077, Novus Biologicals, USA). CTX‐1 concentration in the samples was determined by comparing the absorbance values to a standard curve generated using known concentrations of CTX‐1 standards provided with the kit.

### Nano‐CT Scanning

The tibiae were scanned using a GE V∣TOME∣X M 240 Nano CT scanner at the University of Florida, Gainesville, FL, USA, for high‐resolution X‐ray computed tomography (CT‐scanning). The samples were scanned with a diamond‐tungsten target under the following settings: 75 kV, 150 mA, a 0.5 s detector time, averaging of three images per rotation, and a voxel resolution of 12.4 µm. 3D Slicer software (slicer.org; Brigham & Women's Hospital, Massachusetts, United States) was used to create 3D models of the trabecular network within the distal femur and proximal tibia. The DICOM files were imported, and a label map was created. The segmentation process was automated using a threshold, and manual cleaning of the segments was performed using a smooth crushing tool.

### Immunohistochemistry Staining

The immunohistochemistry (IHC) staining was conducted according to the protocol provided by Abcam (https://www.abcam.com/ps/pdf/protocols/ihc_p.pdf). In brief, 5 µm sections were deparaffinized in fresh xylene, rehydrated in descending alcohol baths, and then subjected to antigen retrieval in 10 mM sodium citrate buffer (pH 6.0) by boiling. To block endogenous hydrogen peroxide, the slides were treated with a hydrogen peroxide blocking reagent (ab64218, Abcam, USA), followed by a protein block (ab64226, Abcam, USA). RANKL (NB100‐56512, Novus Biologicals, USA) was used as a primary antibody for overnight incubation at 4 °C. Goat anti‐Mouse IgG Secondary Antibody [HRP Polymer] (VC001‐025, Novus Biologicals, USA) was used as secondary antibody. The SignalStain DAB Substrate Kit (#8059; Cell Signaling Technology, USA) was utilized for visualizing the reactions. The slides were counterstained with hematoxylin, dehydrated in ascending alcohol baths, cleared in xylene, and coverslipped using Fisher Chemical Permount Mounting Medium (SP15‐100, Fisher Scientific, USA). Sections were visualized using an inverted phase microscope (BZ‐X800E, Keyence, USA).

### Rat Cytokine Array

Blood samples were collected from the animals via cardiac puncture and the serum was then separated for cytokine analysis using the Rat Cytokine Array Q282 (QAR‐CAA‐282, RayBiotech Life, GA, USA). Biomarkers showing no variation across all the subjects (i.e., zero‐variance), were excluded from the analysis. All analyses were conducted in the R programming language V4.2.3 (R Core Team 2017). The Pathway/GO over‐representation analyses were implemented with the R package clusterProfiler.^[^ ^251^ ^]^ The Gene Ontology (GO) term enrichment analysis was conducted as “over‐representation analysis”, which used hypergeometric distribution in the subset of differentially expressed biomarkers.

### Adiposity Evaluation via Sudan Black B (SBB) Staining

Sudan black B staining is a common method used to evaluate adipose tissue. Briefly, the SBB staining solution was prepared by dissolving 0.7 g of SBB in 100 mL 70% ethanol and filtering it through a 0.22 µm filter into a new 100 mL volumetric flask. 5 µm thickness from paraffin‐embedded samples (bone or subcutaneous sections) were prepared, deparaffinized, rehydrated, then stained with SBB staining solution for 3 h. The stained tissue sections are then rinsed with 70% isopropyl alcohol and distilled water, counterstained with Nuclear Fast Red (NFR) red (50‐317‐51, Electron Microscopy Sciences, USA), and mounted with VectaMount AQ Mounting Medium (Vector Laboratories, USA). Finally, the slides are visualized using an inverted phase microscope (BZ‐X800E, Keyence, USA) and quantified using ZEN 3.1 software (Zeiss, USA).

### Metagenomics

The samples were processed and analyzed via ZymoBIOMICS Shotgun Metagenomic Sequencing Service for Microbiome Analysis (Zymo Research, Irvine, CA). DNA was extracted using the ZymoBIOMICS−96 MagBead DNA Kit (Zymo Research, Irvine, CA) and an automated platform.

### Shotgun Metagenomic Library Preparation

Genomic DNA samples were profiled with shotgun metagenomic sequencing. Sequencing libraries were prepared with the KAPA HyperPlus Library Preparation Kit (Kapa Biosystems, Wilmington, MA) with up to 100 ng DNA input following the manufacturer's protocol using internal single‐index 8 bp barcodes with TruSeq adapters (Illumina, San Diego, CA). All libraries were quantified with TapeStation (Agilent Technologies, Santa Clara, CA) and then pooled in equal abundance. The final pool was quantified using qPCR. The final library was then sequenced on either the Illumina HiSeq or the Illumina NovaSeq. Raw sequence reads were trimmed to remove low quality fractions and adapters with Trimmomatic‐0.33:^[^ ^252^ ^]^ quality trimming by sliding window with 6 bp window size and a quality cutoff of 20, and reads with size lower than 70 bp were removed. Microbial composition was profiled with Centrifuge^[^ ^253^ ^]^ using bacterial, viral, fungal, mouse, and human genome datasets. Strain‐level abundance information was extracted from the Centrifuge outputs and further analyzed: 1) to perform alpha‐ and beta‐diversity analyses; 2) to create microbial composition barplots with QIIME;^[^ ^254^ ^]^ and 3) to create taxa abundance heatmaps with hierarchical clustering (based on Bray‐Curtis with default settings (p>0.05 and LDA effect size >2).

### Statistical Analysis

All assays were presented as mean ± standard deviation (SD). Statistical analysis was carried out using GraphPad Prism (version 8.0, US) and groups compared using the nonparametric Mann‐Whitney test. *p* values < 0.05 were considered significant.

### Ethics Approval

No patients were investigated in this study. However, animals were utilized, and all animal procedures were approved by the Institutional Animal Care and Use Committee at the University of Central Florida (IACUC protocol: 2023–24). All procedures were carried out following the guidelines of the American Veterinary Medical Association.

## Conflict of Interest

The authors declare no conflict of interest.

## Author Contributions

All authors made substantial contributions to conception and design, acquisition of data, or analysis and interpretation of data.

## Supporting information

Supporting Information

## Data Availability

The data that support the findings of this study are available from the corresponding author upon reasonable request.
