# Supplementary material for: A Multifunctional Therapeutic Strategy Using P7C3 as A Countermeasure Against Bone Loss and Fragility in An Ovariectomized Rat Model of Postmenopausal Osteoporosis
Source: Adv Sci (Weinh). 2024 Mar 13;11(21):2308698. doi: 10.1002/advs.202308698 (PMC11151083; doi:10.1002/advs.202308698)
Supplement: Supplementary file 1 — Supporting Information [file ADVS-11-2308698-s001.pdf]

ADVANCED SCIENCE
Open Access

Supporting Information

for *Adv. Sci.*, DOI 10.1002/advs.202308698

A Multifunctional Therapeutic Strategy Using P7C3 as A Countermeasure Against Bone Loss and Fragility in An Ovariectomized Rat Model of Postmenopausal Osteoporosis

*Fei Wei, Megan Hughes, Mahmoud Omer, Christopher Ngo, Abinaya Sindu Pugazhendhi, Elayaraja Kolanthai, Matthew Aceto, Yasmine Ghattas, Mehdi Razavi, Thomas J Kean, Sudipta Seal and Melanie Coathup**

Supplementary Information

A Novel Multifunctional Therapeutic Strategy using P7C3 as a Countermeasure Against Bone Loss and Fragility in an Ovariectomized Rat Model of Postmenopausal Osteoporosis

Fei Wei, Megan Hughes, Mahmoud Omer, Christopher Ngo, Matthew Aceto, Yasmine Ghattas, Abinaya Sindu Pugazhendhi, Thomas Kean, Mehdi Razavi, Sudipta Seal, Melanie Coathup

Supplementary Figure S1

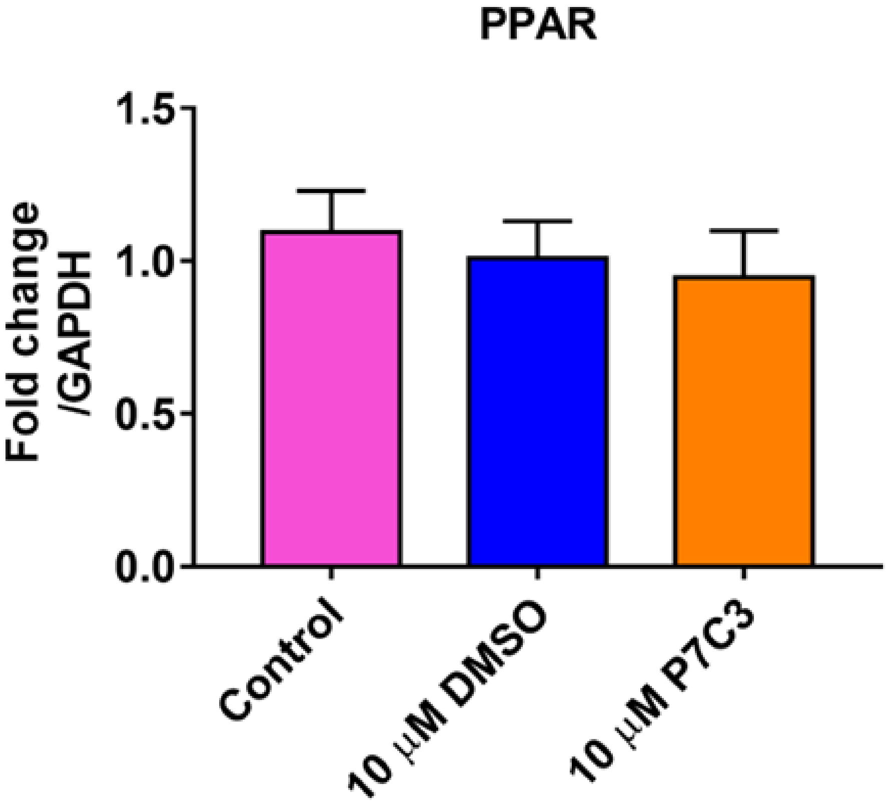

Supplementary Figure S1. RT-qPCR analysis of *PPARγ* gene expression *in vitro*. PPAR*γ* is a transcription factor implicated in adipocyte differentiation and adipocyte maintenance.

Results demonstrate that at this time point and dose, P7C3 did not significantly reduce *PPARγ* expression.

Supplementary Figure S2

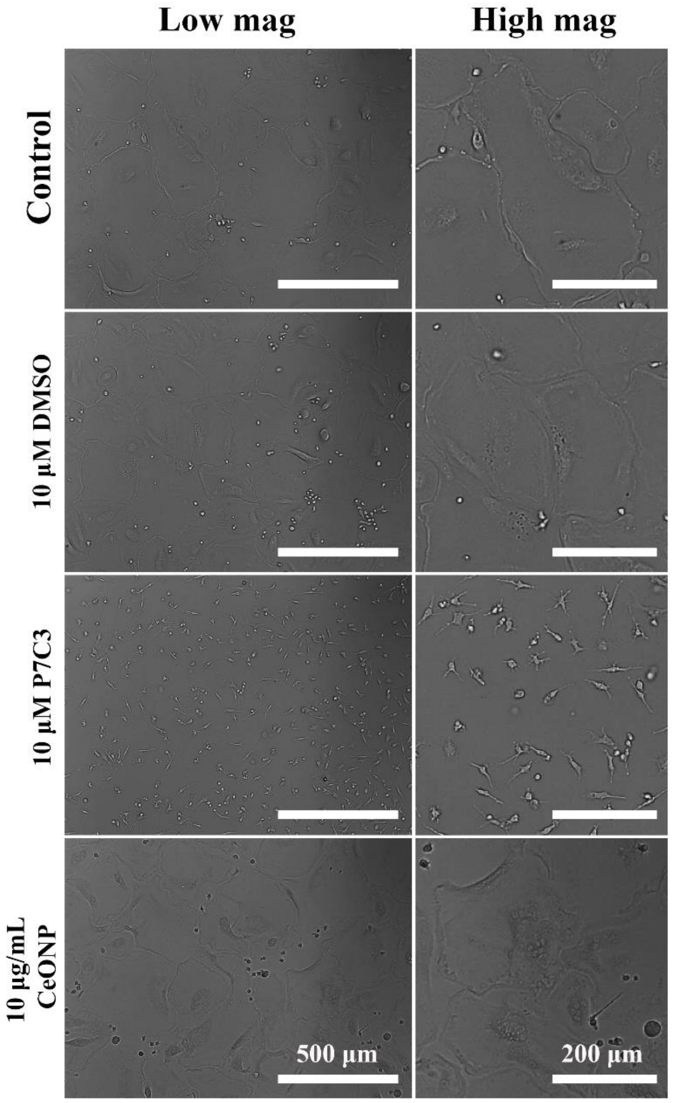

Supplementary Figure S2. P7C3 attenuates osteoclastic activity and its formation *in vitro*. Representative phase contrast images of human osteoclasts precursors cultured with either DMSO or P7C3 over a 6-day period. Cells visualized using an inverted phase microscope.

Supplementary Figure S3

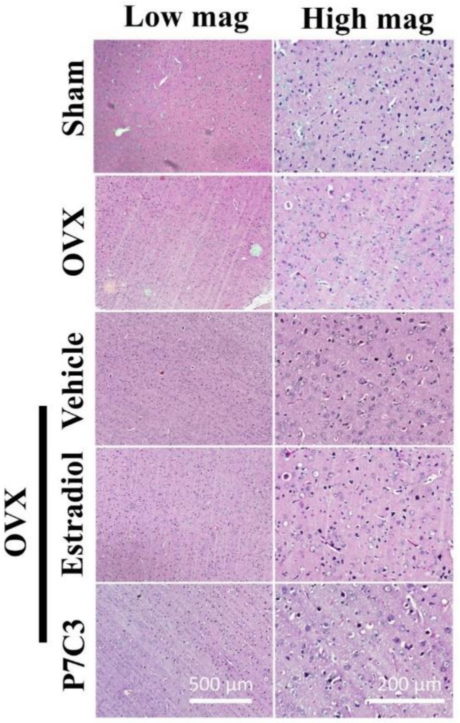

Supplementary Figure S3. Representative H&E staining images of brain sections at 13 weeks post-injection. The images indicate no significant pathological changes were observed.

Supplementary Figure S4

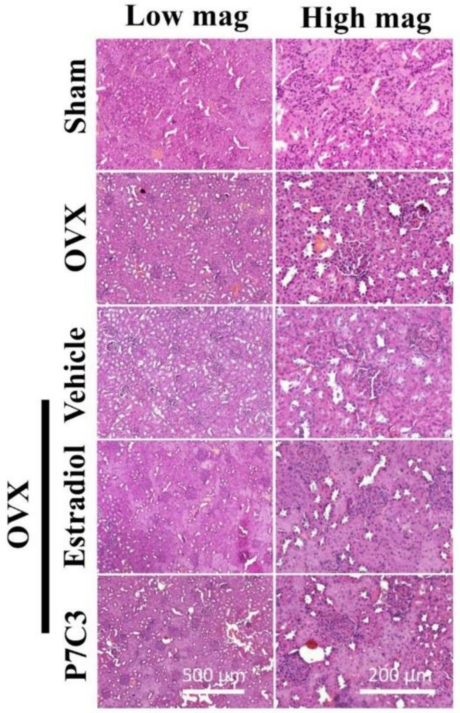

Supplementary Figure S4. Histological analysis of H&E-stained kidney sections. The images indicate that no significant pathological changes were observed in the kidney tissues of any of the treatment groups.

Supplementary Figure S5

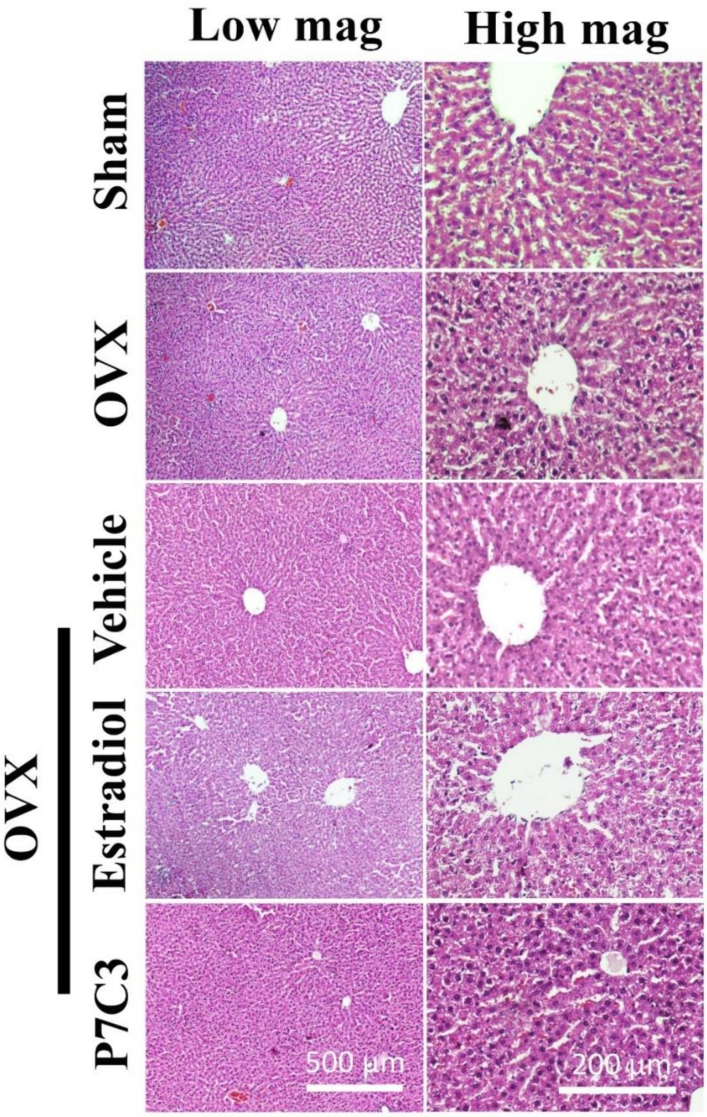

Supplementary Figure S5. Representative H&E-stained liver images in sham and OVX groups after treatment with different agents. There were no significant pathological changes observed in the liver tissue.

Supplementary Figure S6

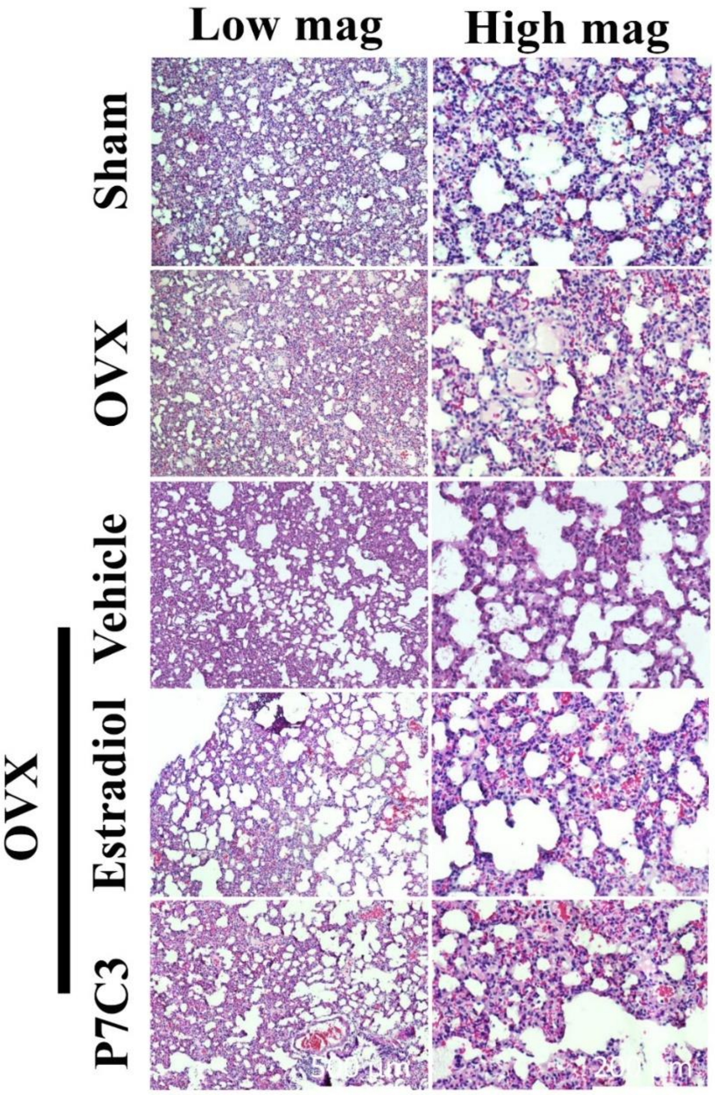

Supplementary Figure S6. Histological analysis of H&E-stained lung sections. The lungs were collected for histological analysis. The image indicates that no significant pathological changes were detected in the lungs.

Supplementary Figure S7

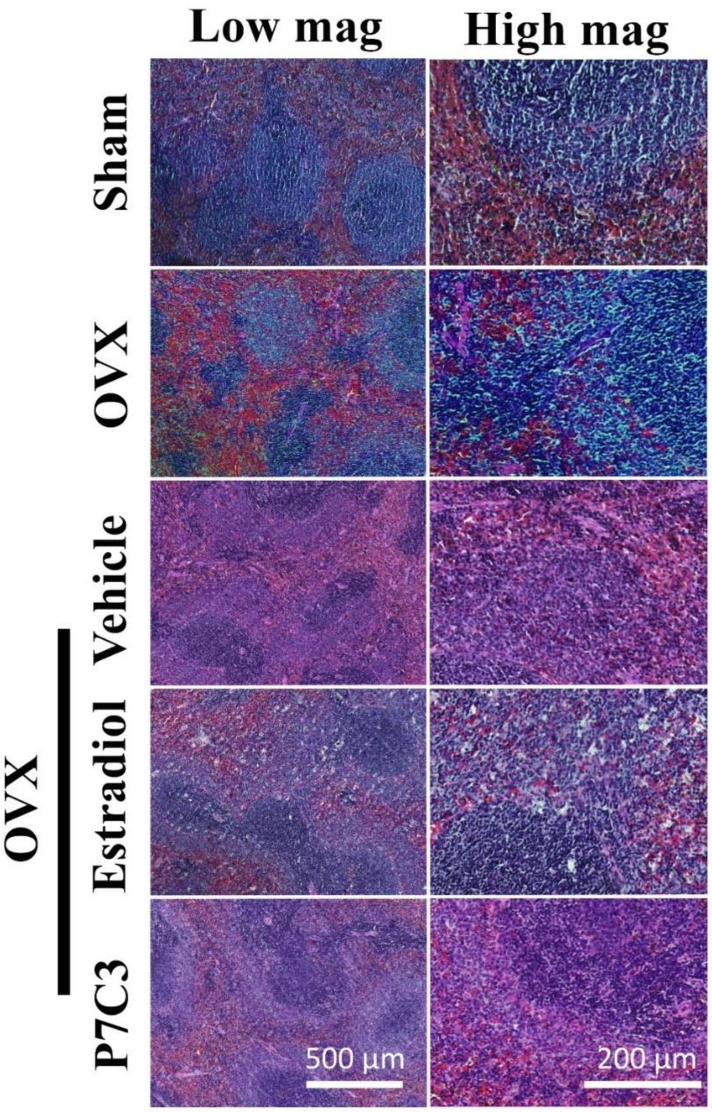

Supplementary Figure S7. Representative H&E staining images of spleen sections from all experimental groups. There were no significant pathological changes observed among the groups.

Supplementary Figure S8

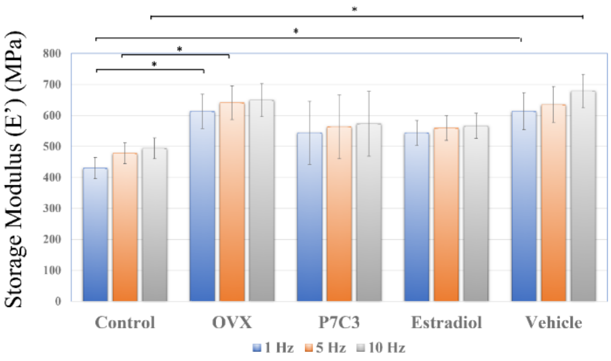

Supplementary Figure S8. The storage modulus (E') indicates how the specimen stores elastic energy, and therefore the 'solid' or hydroxyapatite component within the bone tissue. When storage modulus E' (elastic energy) was examined in each group, and at increasing frequencies of 1, 5, and 10 Hz, a trend was seen where E' increased with increasing frequency. It is generally accepted that within the lower frequency range (e.g., 1 Hz), there is greater movement of the organic component of bone, with less movement of this fraction as the frequency increases. A significantly higher E', and thus elastic component was measured in the OVX group (1 Hz = 613.25 ± 55.36 MPa; 5 Hz = 641.05 ± 54.23 MPa; $p < 0.05$ in both cases), and vehicle control group (1 Hz = 613.65 ± 60.23 MPa; 10 Hz = 678.82 ± 53.28 MPa; $p < 0.05$ in both cases) when compared with healthy control bone tissue. No other significant differences were found. These results suggest a trend where the micro-tissue elastic properties of animals in the OVX and vehicle groups were significantly stiffer (i.e., less movement within the organic component), when compared to control, P7C3 and estradiol given animals. Thus, microcrack formation and rapid propagation are more likely to occur.

Supplementary Figure S9

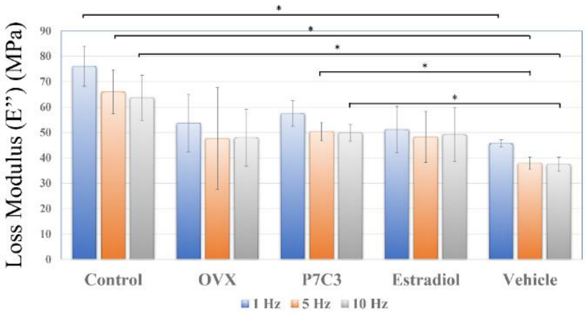

Supplementary Figure S9. The loss modulus (E'') indicates the properties involved in energy dissipation, and therefore the micro-scale viscous properties (e.g., type-I collagen, proteins, and water component) of bone tissue. Results showed a trend where viscosity was highest in healthy control animals when compared with all other groups. Notably, viscosity was significantly higher in the control group at 1 Hz (76.07 ± 7.84 MPa), 5 Hz (65.98 ± 8.69 MPa), and 10 Hz (65.98 ± 8.69 MPa) when compared with the DMSO group (1 Hz 45.64 ± 1.36 MPa, 5 Hz 37.86 ± 2.35 MPa, and 10 Hz 37.42 ± 2.81 MPa; $p < 0.05$ in all cases). Further, a significant increase in viscosity was measured in the P7C3 group at 5 (50.34 ± 3.45 MPa), and 10 Hz (49.88 ± 3.27 MPa) when compared to the vehicle control group ($p < 0.05$ in all cases). No other significant differences were found. These results show a trend where the viscosity of bone in animals in the healthy control group was more ductile, suggesting alterations in the organic matrix in all other groups. These organic alterations altered the mechanical performance of bone tissue on the microscale. Notably, P7C3, and when compared to its vehicle control, significantly reduced the loss in viscosity.

Supplementary Figure S10

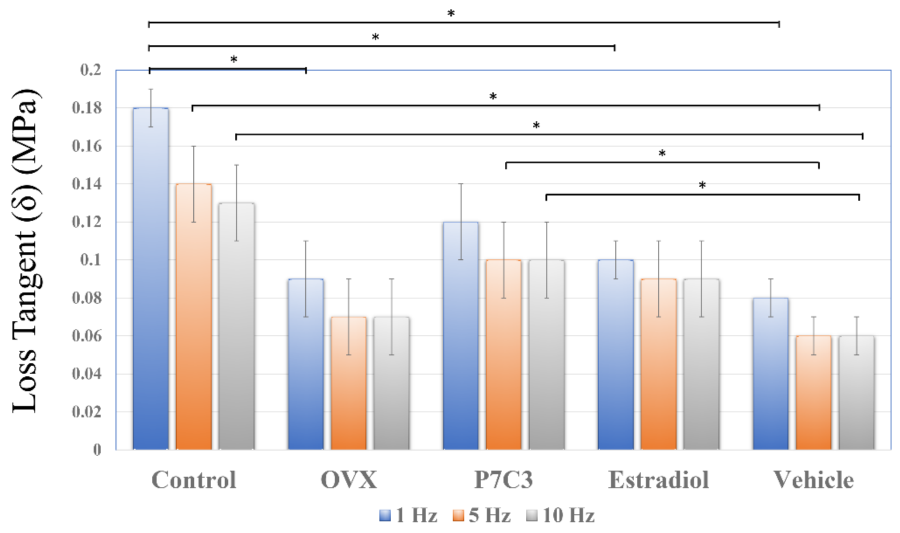

Supplementary Figure S10. Toughness is characteristic of the ability of bone to absorb energy without fracturing, and bone with a low loss tangent, is considered less able to dampen incoming energies, and as such, bone that is less tough, is at a higher risk for microfracture. Our results reveal a significant reduction in loss tangent in the OVX (1 Hz, 0.09 ± 0.02 MPa), estradiol (1 Hz, 0.10 ± 0.01 MPa), and vehicle (1 Hz, 0.09 ± 0.01 MPa) groups when compared

with healthy control animals (1 Hz, 0.18 ± 0.01 MPa; $p < 0.05$ in all cases). Further, a significant decrease in loss tangent was measured in the vehicle group at 5 (0.06 ± 0.01 MPa), and 10 Hz (0.06 ± 0.01 MPa), when compared with healthy control (5 Hz, 0.14 ± 0.02 MPa, 10 Hz 0.13 ± 0.02 MPa). Notably, P7C3 treated animals displayed a significantly increased loss tangent at 5 (0.10 ± 0.02 MPa) and 10 Hz (0.10 ± 0.02 MPa) when compared with its vehicle control group ($p < 0.05$ in both cases). No other significant differences were found. These results show a trend where the ability of bone tissue to dampen incoming energies was reduced compared to healthy control animals, and that P7C3, when compared to its vehicle control, significantly increased tissue toughness, and potentially, its risk to microfracture.

Supplementary Figure S11

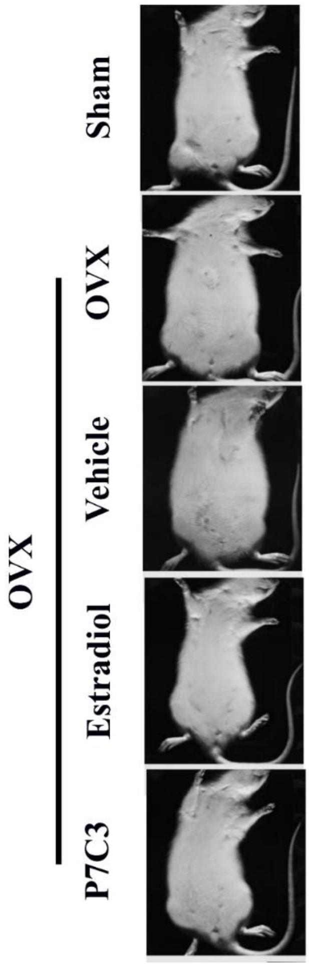

Supplementary Figure S11. Representative gross body mass images of different groups. The OVX and vehicle groups had higher body mass compared to the sham group, while a

noticeable decrease in body size was observed in the Estradiol and P7C3 groups after imaging with a Bruker *In-Vivo* Xtreme unit.

Supplementary Figure S12

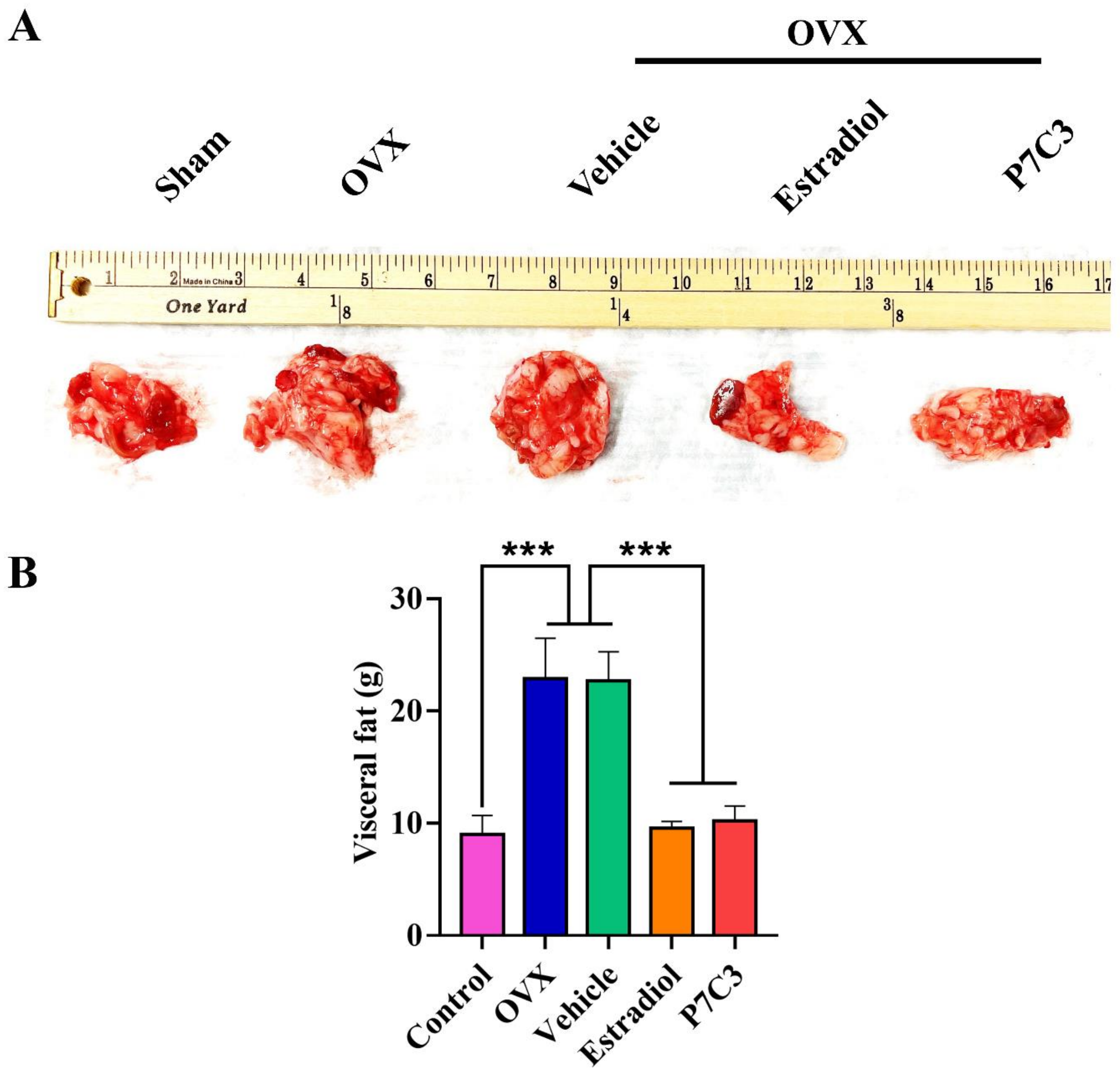

Supplementary Figure S12. Measurement of visceral adipose tissues. [A] Representative gross images of visceral adipose tissue images obtained from the sham, OVX, vehicle, Estradiol, and P7C3 groups. [B] Adipocyte weight of the visceral adipose tissue. Both Estradiol and P7C3 groups demonstrated a significant reduction in the weight of visceral adipose tissue compared to the OVX and vehicle groups ($p < 0.001$).

Supplementary Figure S13

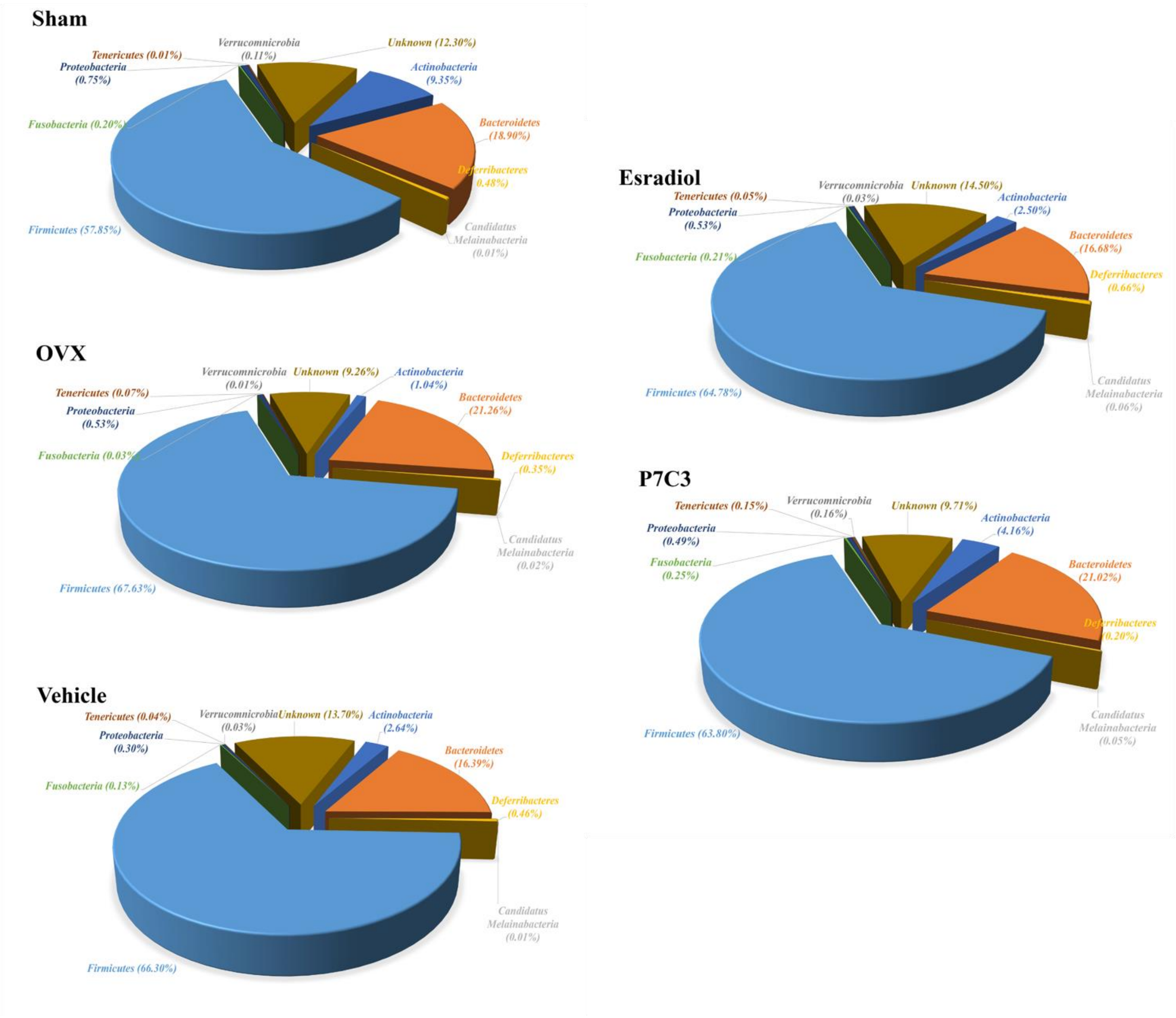

Supplementary Figure S13. Graphical representation showing the abundance of bacteria identified within the GM according to phyla. Actinobacteria, Bacteroidetes, Candidatus Melainabacteria, Deferribacteres, Firmicutes, Fusobacteria, Proteobacteria, Tenericutes, Verrucomicrobia, and unknown, were quantified in each group.

Supplementary Table 1

	Sham		OVX		Vehicle		Estradiol		P7C3	
	Mean (%)	SE (%)	Mean (%)	SE (%)	Mean (%)	SE (%)	Mean (%)	SE (%)	Mean (%)	SE (%)
Actinobacteria	9.346	4.19	1.04	0.21	2.64	0.84	2.5	0.84	4.16	1.7
Bacteroidetes	18.899	3.349	21.26	7.13	16.39	1.49	16.68	0.81	21.02	3.72
Candidatus Melainabacteria	0.011	0.011	0.02	0.01	0.01	0.007	0.06	0.02	0.05	0.02
Deferribacteres	0.481	0.239	0.35	0.08	0.46	0.08	0.66	0.21	0.2	0.08
Firmicutes	57.85	3.62	67.63	6.34	66.3	0.44	64.78	1.35	63.8	3.77
Fusobacteria	0.2	0.107	0.03	0.02	0.13	0.07	0.21	0.08	0.25	0.09
Proteobacteria	0.748	0.225	0.53	0.07	0.3	0.05	0.53	0.03	0.49	0.14
Tenericutes	0.098	0.033	0.07	0.01	0.04	0.008	0.05	0.01	0.15	0.03
Verrucomnicrobia	0.109	0.046	0.01	0.01	0.03	0.01	0.03	0.03	0.16	0.11
Unknown	12.3	4.09	9.26	0.92	13.7	1.3	14.5	0.38	9.71	1.4

Supplementary Table 1. Measurement of the abundance of bacteria identified within the GM according to phyla. The percentage abundance of the various microbiota within the GM in each of the experimental groups was investigated. Data is presented as mean and standard error.

Supplementary Figure S14

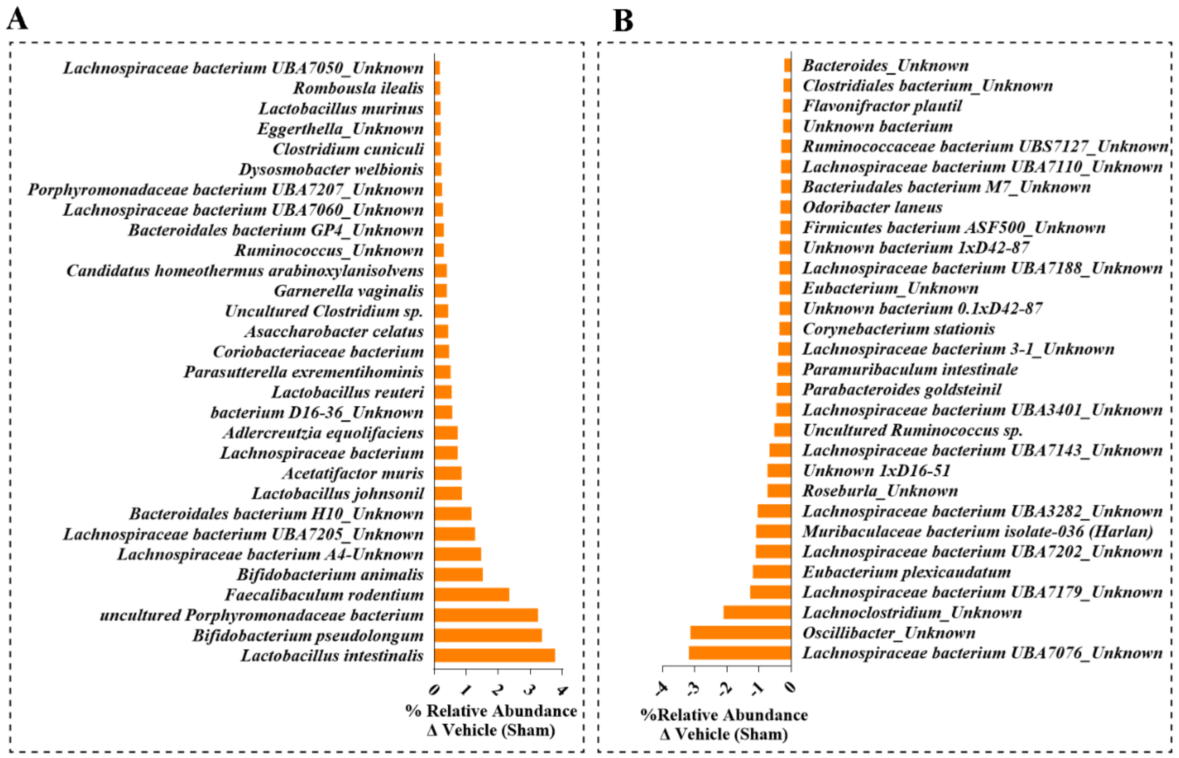

Supplementary Figure S14. The top 30 up- and down-regulated bacterial species in the GM. Levels of DNA abundance were analyzed and the top 30 upregulated [A], and downregulated [B] species in response to treatment in the DMSO-vehicle control group are presented.
